# Liposomal Morin Attenuates DMH-Associated Colonic Oxidative Stress, Inflammation, and Apoptosis/Autophagy-Related Dysregulation in Rats

**DOI:** 10.3390/ph19091400

**Published:** 2026-09-04

**Authors:** Mohammed A. Akeel, Ekramy M. Elmorsy, Fahad M. Alshammari, Aly A. M. Shaalan, Abdulrahman S. Aldaghmi, Barakat M. Alrashdi, Saad M. Alrashidi, Gehad E. Elshopakey, Baraah Abu Alsel, Manal S. Fawzy

**Affiliations:** 1 Department of Human Anatomy, Faculty of Medicine, Jazan University, Jazan 45142, Saudi Arabia; m.akeel@jazanu.edu.sa (M.A.A.); ashaalan@jazanu.edu.sa (A.A.M.S.); 2 Center for Health Research, Northern Border University, Arar 73213, Saudi Arabia; ekramy.elmorsy@nbu.edu.sa; 3 Department of Biology, College of Science, Jouf University, Sakaka 72341, Aljouf, Saudi Arabia; fahadm@ju.edu.sa (F.M.A.); asaldaghmi@ju.edu.sa (A.S.A.); 4 Department of Histology and Cell Biology, Faculty of Medicine, Suez Canal University, Ismailia 41522, Egypt; 5 Radiation and Oncology Comprehensive Cancer Center, King Fahad Medical City, College of Medicine, Alfaisal University, Riyadh 11525, Saudi Arabia; saadmrashidi@gmail.com; 6 Department of Clinical Pathology, Faculty of Veterinary Medicine, Mansoura University, Mansoura 35516, Egypt; gehadelshopakey@mans.edu.eg; 7 Medical Sciences & Preparatory Year Department, North Private College of Nursing, Arar 73244, Saudi Arabia; baraahsel@nec.edu.sa

**Keywords:** morin, liposomes, nanotechnology, colorectal carcinogenesis, oxidative stress, inflammation, apoptosis, autophagy, NRF2/HO-1, NF-κB/COX-2

## Abstract

**Background/Objectives**: Oxidative stress, chronic inflammation, disrupted apoptosis, and altered autophagy are biological processes implicated in colorectal tumor development. Morin is a plant-derived flavonoid with antioxidant and anti-inflammatory properties, but its limited solubility and bioavailability may limit its biological activity. This study evaluated the effects of free morin and morin-loaded liposomes (MOR-Lips) on 1,2-dimethylhydrazine (DMH)-associated colonic biochemical, molecular, and histopathological alterations in rats. **Methods**: Rats were randomly assigned to six groups—vehicle control, MOR, MOR-Lips, DMH, DMH + MOR, and DMH + MOR-Lips—and treated for 10 weeks. Serum and colonic tissues were evaluated for cancer-associated biomarkers (CEA, CA19-9, CA125, HMG-CoA reductase), oxidative stress and antioxidant indices, nitrosative and oxidative DNA-damage markers (MDA, NO, 8-OHdG), inflammatory mediators (TLR4/NF-κB/COX-2, cytokines, MPO), proliferative indices (Ki-67, PCNA), apoptotic and autophagy-related regulators (Bax, caspase-3, p53, cytochrome c, BCL-2, p-AKT, LC3-II, Beclin-1, p62), and histopathological and immunohistochemical changes. **Results**: DMH exposure was associated with increased CEA, CA19-9, CA125, HMG-CoA reductase, MDA, NO, 8-OHdG, TLR4/NF-κB/COX-2, cytokines, MPO, Ki-67, and PCNA. DMH also reduced NRF2/HO-1 signaling and antioxidant defenses, shifted apoptosis-related markers toward a pro-survival profile, altered autophagy-related markers, and produced marked colonic histopathological abnormalities. Both free MOR and MOR-Lips attenuated several of these DMH-associated alterations, with MOR-Lips generally producing greater effects than free MOR. MOR-LIP treatment was associated with restoration of antioxidant marker profiles, reduced levels of inflammatory and proliferative markers, a shift toward a pro-apoptotic marker profile, partial normalization of autophagy-related markers, and improved colonic histopathological appearance. **Conclusions**: In DMH-exposed rats, MOR-Lips were associated with more favorable redox, inflammatory, proliferative, apoptosis-related, autophagy-related, and histopathological profiles than free MOR. These findings support further investigation of MOR-Lips as a formulation strategy for improving the biological activity of morin. Because quantitative preneoplastic and neoplastic endpoints were not measured, the results do not establish inhibition of colorectal carcinogenesis or chemopreventive efficacy.

## 1. Introduction

Colorectal cancer (CRC) is among the most common malignancies worldwide, accounting for around 10% of newly diagnosed cancer cases and approximately 9.4% of cancer-related mortality [1]. It is also reported as the second most common cancer globally [2]. Despite advances in diagnostic tools and therapeutic strategies, metastatic CRC remains difficult to treat effectively [3]. Patients with advanced disease continue to show low long-term survival, with 5-year survival rates not exceeding 15% [4]. These observations highlight the need for safe and effective approaches that modulate biological processes implicated in CRC development and progression.

Oxidative stress is a key event in the development and progression of colorectal cancer. Increased metabolic activity in cancer cells promotes excessive generation of reactive oxygen species (ROS), including hydroxyl radicals, the superoxide anion, and hydrogen peroxide [5]. These species arise mainly from mitochondria, but they also originate from the endoplasmic reticulum and peroxisomes [6]. ROS exert dual effects in cells: at low concentrations, they participate in intracellular signaling and support proliferation, whereas uncontrolled ROS generation damages DNA, proteins, and membrane lipids [7]. Oxidative biomarkers such as 8-hydroxy-2′-deoxyguanosine (8-OHdG) and malondialdehyde (MDA) are closely associated with genomic instability, DNA mutations, and the advancement of colorectal cancer [8]. Protection against ROS-induced injury depends largely on endogenous antioxidants such as reduced glutathione (GSH), superoxide dismutase (SOD), and catalase (CAT). The NRF2/HO-1 pathway further strengthens this defense by regulating cellular antioxidant responses and cellular redox homeostasis [9,10].

Inflammation also plays a crucial role in colorectal carcinogenesis and is tightly linked to oxidative stress. Activation of the Toll-like receptor 4 (TLR4)/nuclear factor kappa B (NF-κB)/cyclooxygenase-2 (COX-2) axis results in marked increases in inflammatory mediators, including tumor necrosis factor-alpha (TNF-α), interleukin-1 beta (IL-1β), interleukin-6 (IL-6), and myeloperoxidase (MPO) [11,12,13]. These alterations create a tumor-promoting microenvironment that favors colorectal cancer progression [5]. In addition, CRC development is characterized by disruption of normal cell-cycle control and enhanced cell proliferation. Elevated expression of the proliferation marker Ki-67 and proliferating cell nuclear antigen (PCNA) reflects increased proliferative capacity [14,15], while abnormal activation of AKT signaling enhances cellular survival and supports malignant transformation [16]. In the same context, resistance to apoptosis is a defining feature of CRC progression. Colorectal cancer cells often exhibit reduced expression of B-cell lymphoma 2-associated X protein (Bax), p53, cytochrome c, and caspase-3, accompanied by upregulation of B-cell lymphoma-2 (Bcl-2) [9,17]. This shift disrupts mitochondrial apoptotic signaling and promotes tumor cell persistence [16]. Oxidative stress, inflammation, apoptosis, and autophagy are interconnected processes; dysregulation of one pathway can influence the activity of the others and, together, favor a pro-survival cellular environment [18,19,20,21].

The study of colorectal cancer has greatly benefited from experimental animal models. The 1,2-dimethylhydrazine (DMH)-induced rat model remains one of the most reliable and widely used models because of its strong resemblance to human colorectal cancer [22]. DMH functions as a colon-specific procarcinogen that is metabolically activated in the liver to produce azoxymethane and methylazoxymethanol [22]. These metabolites generate methyldiazonium ions that cause DNA methylation and genetic instability, ultimately driving colorectal tumorigenesis [23]. Accordingly, the DMH model is useful for investigating carcinogenesis-associated colonic alterations and for evaluating the biological effects of candidate interventions. However, biochemical and histopathological findings alone do not establish effects on preneoplastic lesion formation or tumor burden unless these endpoints are quantitatively assessed.

Flavonoids are natural compounds with diverse biological activities, including antioxidant and anti-inflammatory effects [24]. Morin (MOR) is a natural flavonoid present in figs and other plants of the Moraceae family [25]. It has demonstrated antioxidant, antimutagenic, anti-inflammatory, and anticancer-related activities in experimental systems [10,17,26]. However, its biological application may be limited by low aqueous solubility and poor bioavailability [27]. Liposomes are phospholipid-bilayer vesicles that can incorporate hydrophobic compounds such as MOR within their lipid bilayer. Liposomal encapsulation may provide a useful formulation approach to improve the aqueous dispersibility, physicochemical stability, and delivery characteristics of poorly soluble compounds [27,28]. However, the pharmacokinetic, biodistribution, colon-targeting, and safety advantages of a specific liposomal formulation require direct experimental evaluation [29].

In the present study, we evaluated the effects of morin-loaded liposomes (MOR-Lips), compared with free MOR, on DMH-associated biochemical, molecular, and histopathological alterations in rat colonic tissue. The study focused on redox regulation, inflammatory signaling, proliferation-related markers, apoptosis-related markers, autophagy-related markers, and cancer-associated serum biomarkers. Because quantitative preneoplastic and neoplastic endpoints, such as aberrant crypt foci, tumor incidence, and tumor burden, were not assessed, the study was not designed to establish inhibition of colorectal carcinogenesis or chemopreventive efficacy.

## 2. Results

### 2.1. Physicochemical Characterization of MOR-Lips

**Figure 1:**
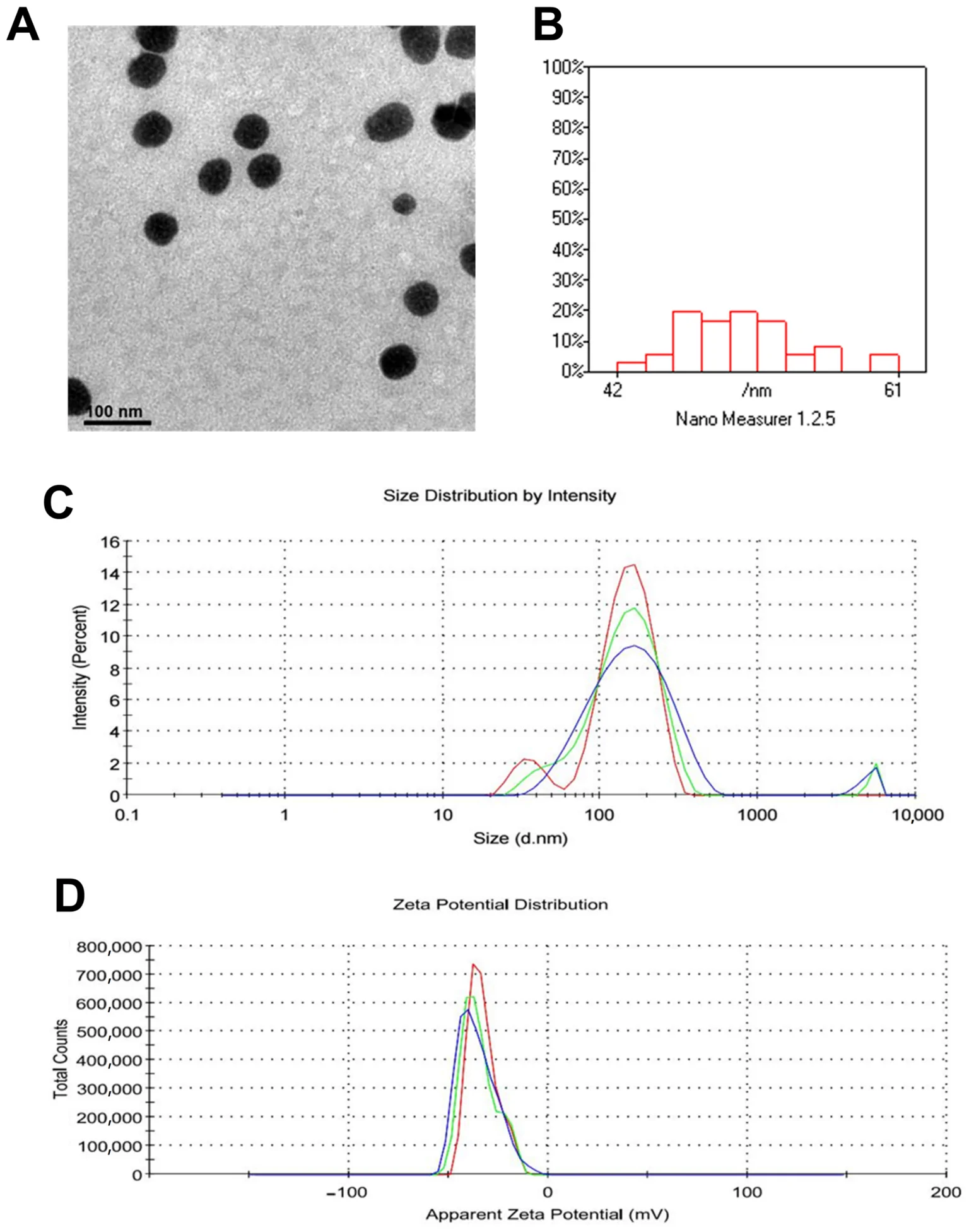
Physicochemical characterization of morin-loaded liposomes (MOR-Lips). (**A**) TEM micrograph showing nearly spherical liposomal vesicles. (**B**) TEM-based particle size histogram indicating that the predominant vesicle population ranges from approximately 42 to 61 nm. (**C**) Intensity-weighted dynamic light scattering size distributions of MOR-Lips. The red, green, and blue traces represent three technical replicate measurement records. The DLS analysis showed a Z-average hydrodynamic diameter of 134.5 nm and a PDI of 0.378, with a predominant intensity peak at approximately 171.5 nm. (**D**) Zeta-potential distribution of MOR-Lips. The red, green, and blue traces represent three technical replicate measurements, showing a consistently negative surface-charge distribution centered at approximately −47.18 mV.

TEM imaging revealed that MOR-Lips were nearly spherical and well dispersed, with no marked visible aggregation in the representative micrograph (Figure 1A). TEM-based image analysis indicated that the predominant vesicle population ranged from approximately 42 to 61 nm (Figure 1B). Dynamic light scattering (DLS) showed a Z-average hydrodynamic diameter of 134.5 nm and a polydispersity index (PDI) of 0.378 (Figure 1C). The intensity-weighted distribution showed a predominant population at approximately 171.5 nm, accounting for 96.2% of the measured intensity. The zeta-potential distribution remained negative, centered at approximately −47.18 mV (Figure 1D).

### 2.2. Encapsulation Efficiency, Drug Loading, and in Vitro Release

MOR-Lips showed high encapsulation efficiency (>80%) and a drug loading capacity of about 7%, indicating efficient incorporation of morin into the lipid bilayer and minimal drug loss during preparation. Together, these parameters support the successful fabrication of a stable, morin-rich liposomal system.

**Figure 2:**
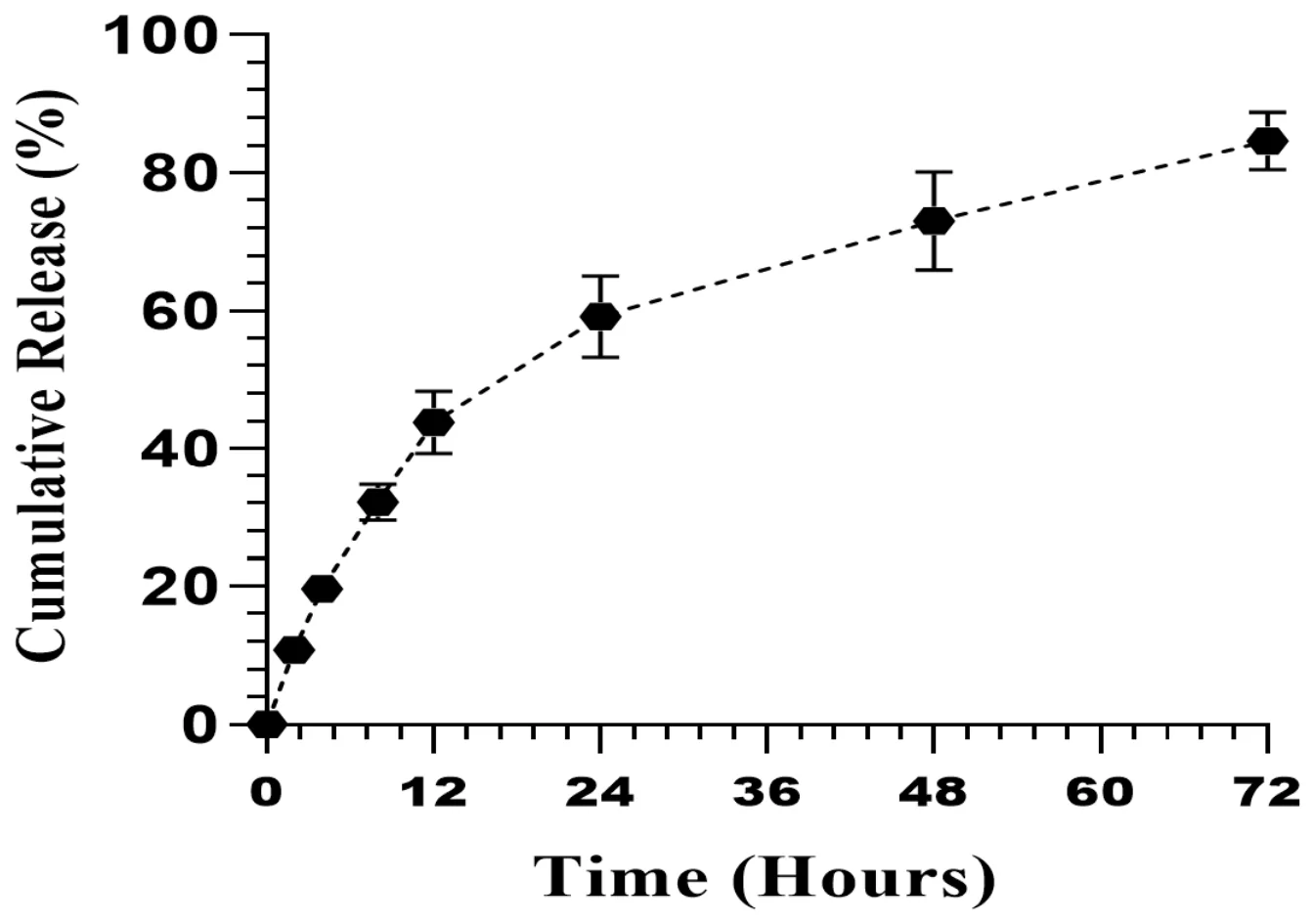
In vitro cumulative release profile of morin from liposomes (MOR-Lips). MOR release was evaluated using a dialysis bag diffusion method in PBS (pH 7.4) containing 0.5% Tween 80 at 37 ± 0.5 °C. The formulation exhibited a biphasic pattern with an initial moderate release phase followed by sustained release up to 72 h. Data are presented as mean ± SD (*n* = 3).

In vitro, MOR-Lips exhibited a biphasic release profile (Figure 2). An initial modest release phase during the first hours was followed by a sustained and gradual increase over 72 h, with approximately 60% of the loaded drug released by 24 h, more than 70% by 48 h, and above 80% by 72 h. This pattern suggests that the liposomal system can provide prolonged morin delivery under physiological-like conditions.

### 2.3. Fourier Transform Infrared Spectroscopy (FTIR) Analysis

**Figure 3:**
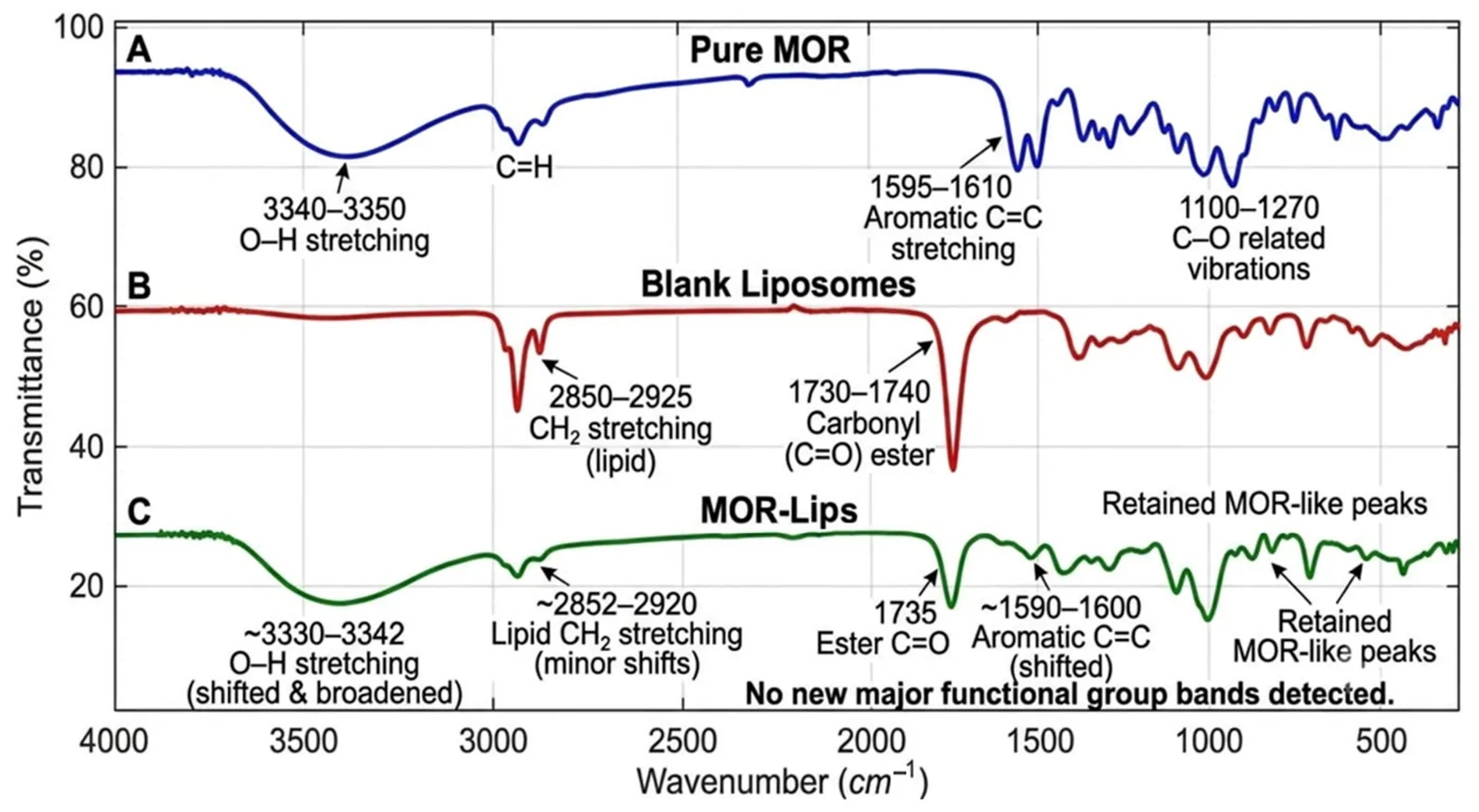
FTIR spectra of pure morin (MOR, **A**), blank liposomes (**B**), and morin-loaded liposomes (MOR-Lips, **C**). Spectra show retention of characteristic MOR and lipid bands with slight shifts and broadening, and no new peaks, consistent with non-covalent interactions and successful physical encapsulation of MOR within the liposomal bilayer.

The FTIR spectrum of pure morin displayed characteristic O–H stretching, aromatic C=C, and C–O bands in the expected regions, while blank liposomes showed typical lipid-associated CH_2_ and ester C=O signals. In MOR-Lips, the principal morin bands were preserved but exhibited slight shifts and broadening, and minor shifts were also observed in lipid-related bands. No novel peaks or marked disappearance of characteristic signals were detected. These features indicate non-covalent interactions between morin and the phospholipid bilayer, supporting successful physical encapsulation without chemical modification (Figure 3).

### 2.4. Stability of MOR-Lips

The storage stability of MOR-Lips was evaluated at 4 °C over 3 months (Table 1). Particle size increased modestly from 65.1 ± 1.8 nm at baseline to 68.3 ± 2.2 nm after 3 months, while PDI changed from 0.182 ± 0.012 to 0.201 ± 0.015. The zeta potential remained negative throughout storage, changing slightly from −47.1 ± 1.5 to −45.4 ± 1.8 mV. Morin retention remained high, decreasing from 100.0% at baseline to 96.2 ± 1.4% after 3 months. Collectively, these results indicate that MOR-Lips retained their nanosized liposomal formulation distribution, surface-charge characteristics, and morin content during refrigerated storage for 3 months.

### 2.5. Effects on Serum Biomarkers and HMG-COA Reductase

**Figure 4:**
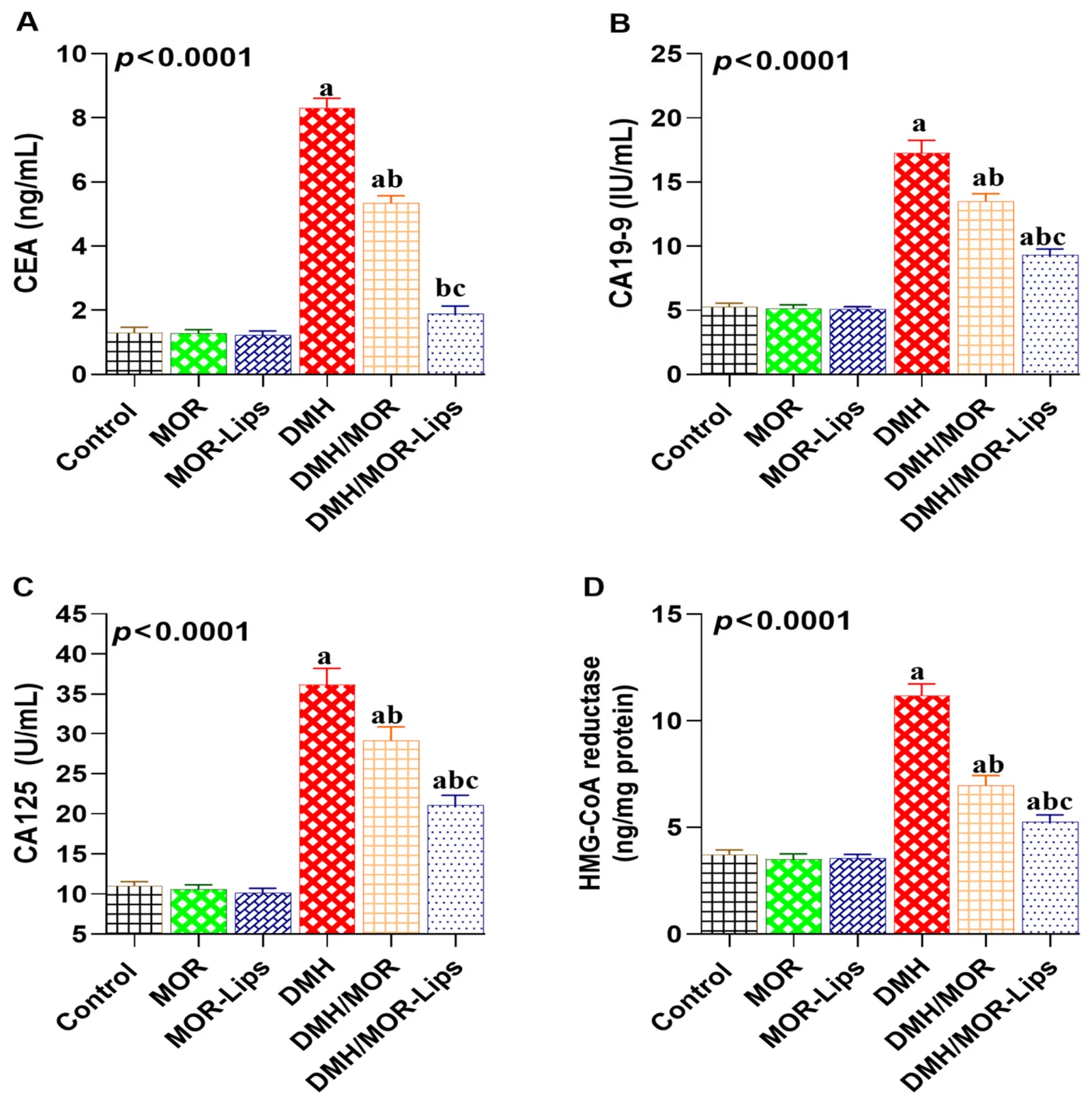
Serum biomarkers and colonic HMG-CoA reductase levels in DMH-induced colon carcinogenesis and the effects of crude and liposomal morin. (**A**) CEA (carcinoembryonic antigen), (**B**) CA19-9 (carbohydrate antigen 19-9), (**C**) CA125 (carbohydrate antigen 125), and (**D**) HMG-CoA reductase. Data are expressed as mean ± SEM. Letters (a–c) indicate significant differences (*p* < 0.05) versus control (a), DMH (b), and crude MOR (c).

DMH administration markedly increased serum CEA, CA19-9, and CA125 compared with controls, confirming the establishment of a tumorigenic state (Figure 4A–C). Treatment with free morin significantly lowered all three markers relative to the DMH group, whereas MOR-Lips produced a more pronounced reduction. Notably, CEA levels in the DMH + MOR-Lips group were restored to values statistically comparable to those of the control group, while CA19-9 and CA125, although substantially improved, remained slightly elevated.

In colonic tissue, DMH markedly activated the mevalonate pathway, as reflected by a robust increase in HMG-CoA reductase levels (Figure 4D). Both morin formulations significantly reduced this DMH-induced rise, with MOR-Lips achieving a greater inhibition than free morin. Enzyme levels in the DMH + MOR-Lips group approached those of the control group, whereas values in the DMH + MOR group remained significantly higher.

### 2.6. Effects on NRF2 Signaling, Antioxidant Defenses, and Oxidative Stress Markers

**Figure 5:**
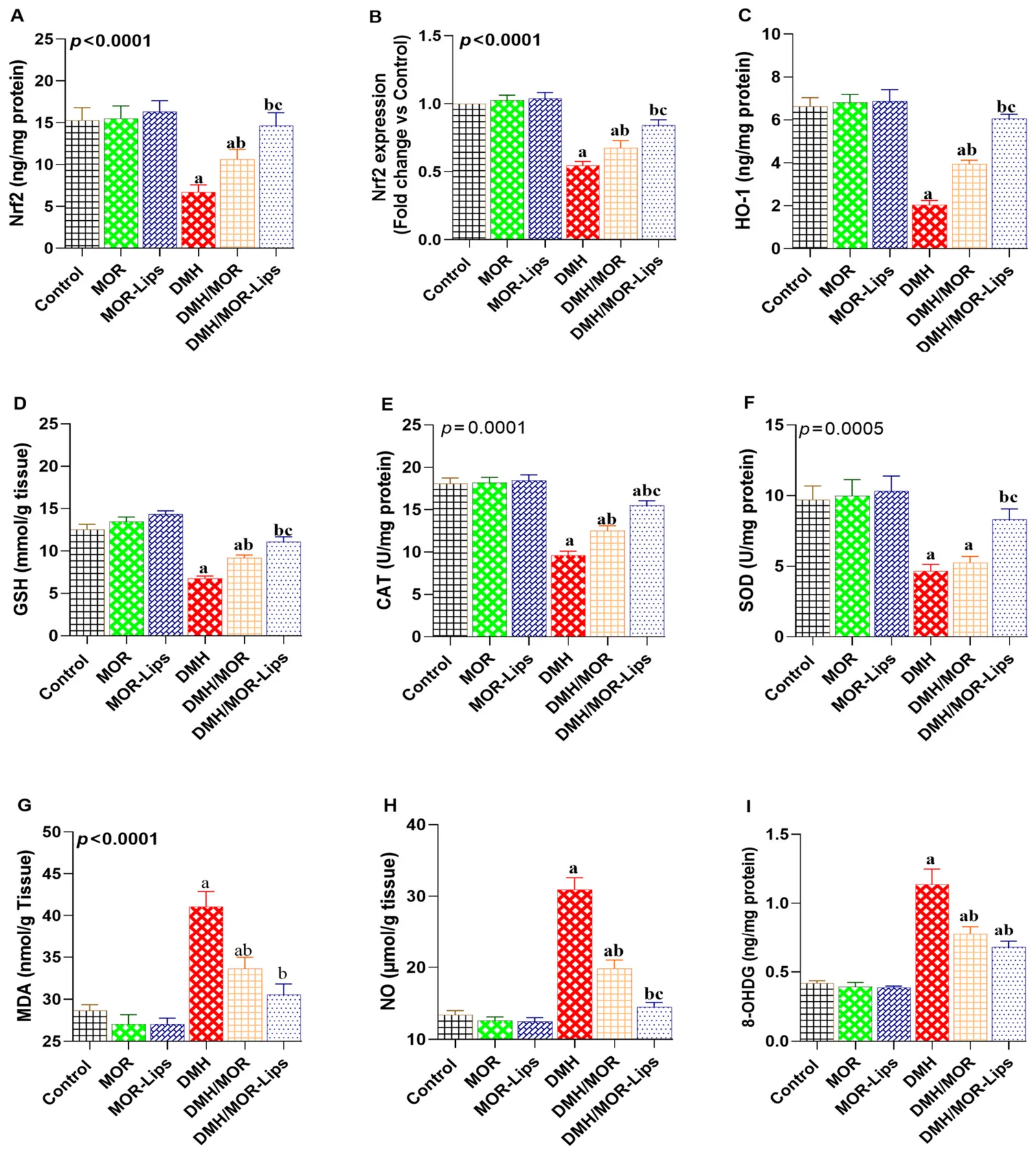
Effect of Crude and Liposomal Morin on NRF2 Signaling, Antioxidant Defense System, and Oxidative Stress Markers in DMH-Induced Colon Carcinogenesis. (**A**) NRF2 (Nuclear Factor Erythroid 2–Related Factor 2) protein expression, (**B)** NRF2 (Nuclear Factor Erythroid 2–Related Factor 2) mRNA expression, (**C**) HO-1 (Heme Oxygenase-1), (**D**) GSH (Reduced Glutathione), (**E**) CAT (Catalase), (**F**) SOD (Superoxide Dismutase), (**G**) MDA (Malondialdehyde), (**H**) NO (Nitric Oxide), (**I**) 8-OHdG (8-Hydroxy-2′-Deoxyguanosine). Mean ± SEM is used to present the results; annotations (a–c) indicate significant differences (*p* < 0.05) when compared with control (a), DMH (b), and crude MOR (c).

DMH exposure profoundly perturbed colonic redox homeostasis (Figure 5A–C). NRF2 protein and mRNA expression, as well as HO-1 levels, were significantly suppressed compared with controls. Free morin partially restored NRF2/HO-1, while MOR-Lips produced a more robust recovery, normalizing NRF2 protein and HO-1 expression to near-control values and markedly enhancing NRF2 transcript levels.

Consistent with these changes, DMH significantly depleted GSH, SOD, and CAT and increased MDA, NO, and 8-OHdG (Figure 5D–I). Both morin formulations improved antioxidant status and attenuated oxidative stress. MOR-Lips were particularly effective in restoring CAT and SOD activities and in reducing NO levels, bringing NO and MDA back to control-comparable values. For MDA and 8-OHdG, both treatments provided similar protection.

### 2.7. Effects on TLR4/NF-κB/COX-2 Signaling and Pro-Inflammatory Mediators

**Figure 6:**
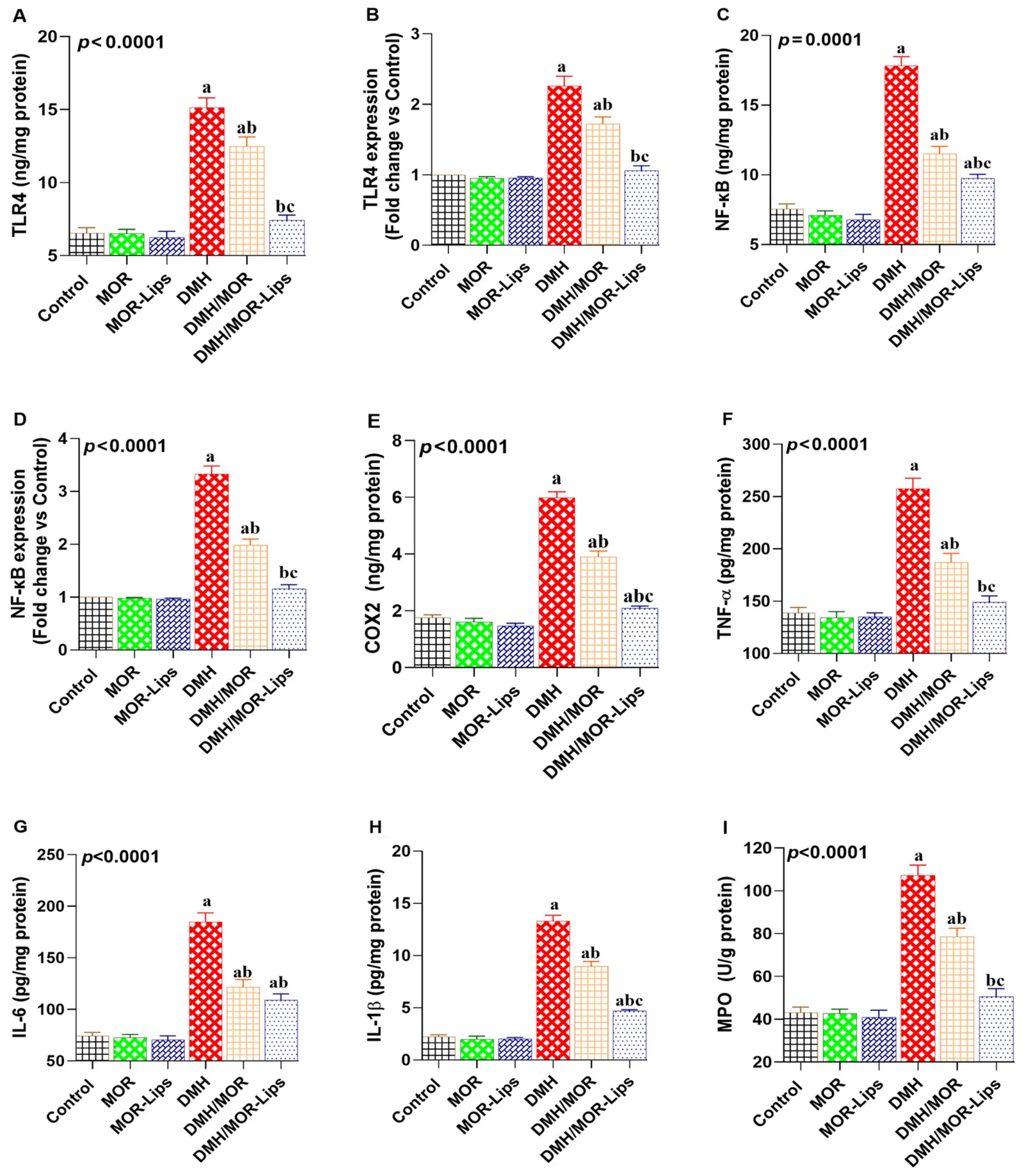
Effect of Crude and Liposomal Morin on TLR4/NF-κB/COX-2 Signaling Pathway, Pro-inflammatory Cytokines, and MPO Levels in DMH-Induced Colon Carcinogenesis. (**A**,**B**) TLR4 (Toll-Like Receptor 4) protein and mRNA expression, (**C**,**D**) NF-κB (Nuclear Factor kappa B) protein and mRNA expression, (**E**) COX-2 (Cyclooxygenase-2), (**F**) TNF-α (Tumor Necrosis Factor-α), (**G**) IL-6 (Interleukin-6), (**H**) IL-1β (Interleukin-1 beta), (**I**) MPO (Myeloperoxidase). Mean ± SEM is used to present the results; annotations (a–c) indicate significant differences (*p* < 0.05) when compared with control (a), DMH (b), and crude MOR (c).

DMH strongly activated the TLR4/NF-κB/COX-2 axis at both protein and transcript levels (Figure 6A–E), indicating pronounced inflammatory signaling in colonic tissue. Free morin significantly reduced TLR4, NF-κB, and COX-2 expression, whereas MOR-Lips induced a deeper suppression, especially for TLR4 (protein and mRNA), NF-κB mRNA, and COX-2. In the MOR-Lips group, these parameters returned to levels that did not differ significantly from controls, except for NF-κB protein, which remained mildly elevated.

DMH also caused marked increases in TNF-α, IL-6, IL-1β, and MPO (Figure 6F–I). Co-treatment with morin attenuated all of these inflammatory mediators, with MOR-Lips again showing stronger effects than free morin on TNF-α, IL-1β, and MPO. TNF-α and MPO were normalized to control-like levels in the DMH + MOR-Lips group, while IL-6 and IL-1β, though markedly decreased, remained modestly higher than in controls.

### 2.8. Effects on Proliferative Markers

**Figure 7:**
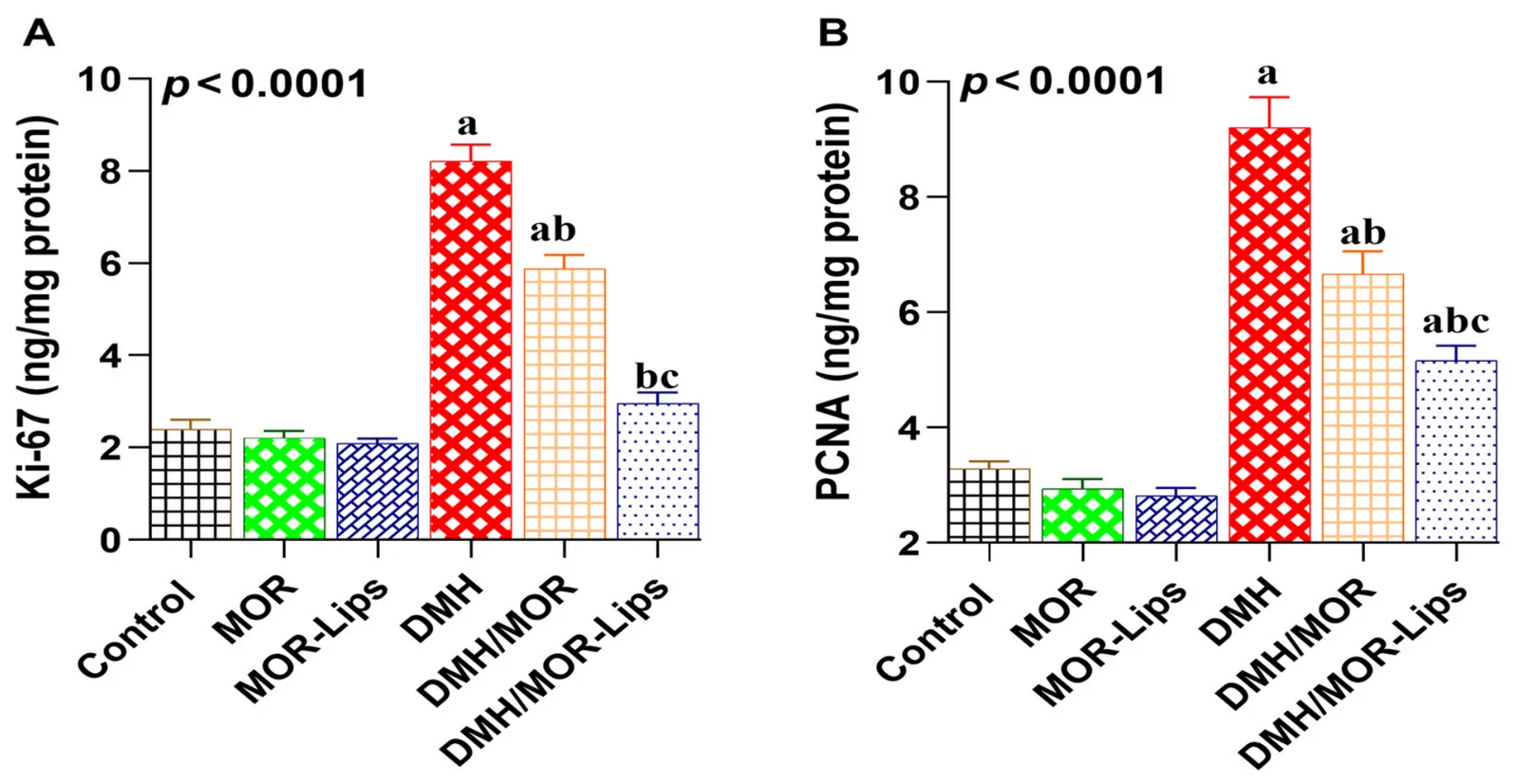
Effect of Crude and Liposomal Morin on Proliferative Activity in Colonic Tissue in DMH-Induced Colon Carcinogenesis. (**A**) Ki-67 (Marker of Cell Proliferation), (**B**) PCNA (Proliferating Cell Nuclear Antigen). Mean ± SEM is used to present the results; annotations (a–c) indicate significant differences (*p* < 0.05) when compared with control (a), DMH (b), and crude MOR (c).

DMH induction markedly enhanced colonic proliferative activity, as indicated by significant increases in Ki-67 and PCNA levels compared with the normal control group (Figure 7A,B). Free morin co-treatment significantly reduced both markers relative to DMH alone, whereas MOR-Lips exerted stronger antiproliferative effects. Ki-67 levels in the DMH + MOR-Lips group were restored to control-comparable values, while PCNA remained slightly but significantly elevated despite substantial improvement.

### 2.9. Effects on Apoptotic and Survival-Related Markers

**Figure 8:**
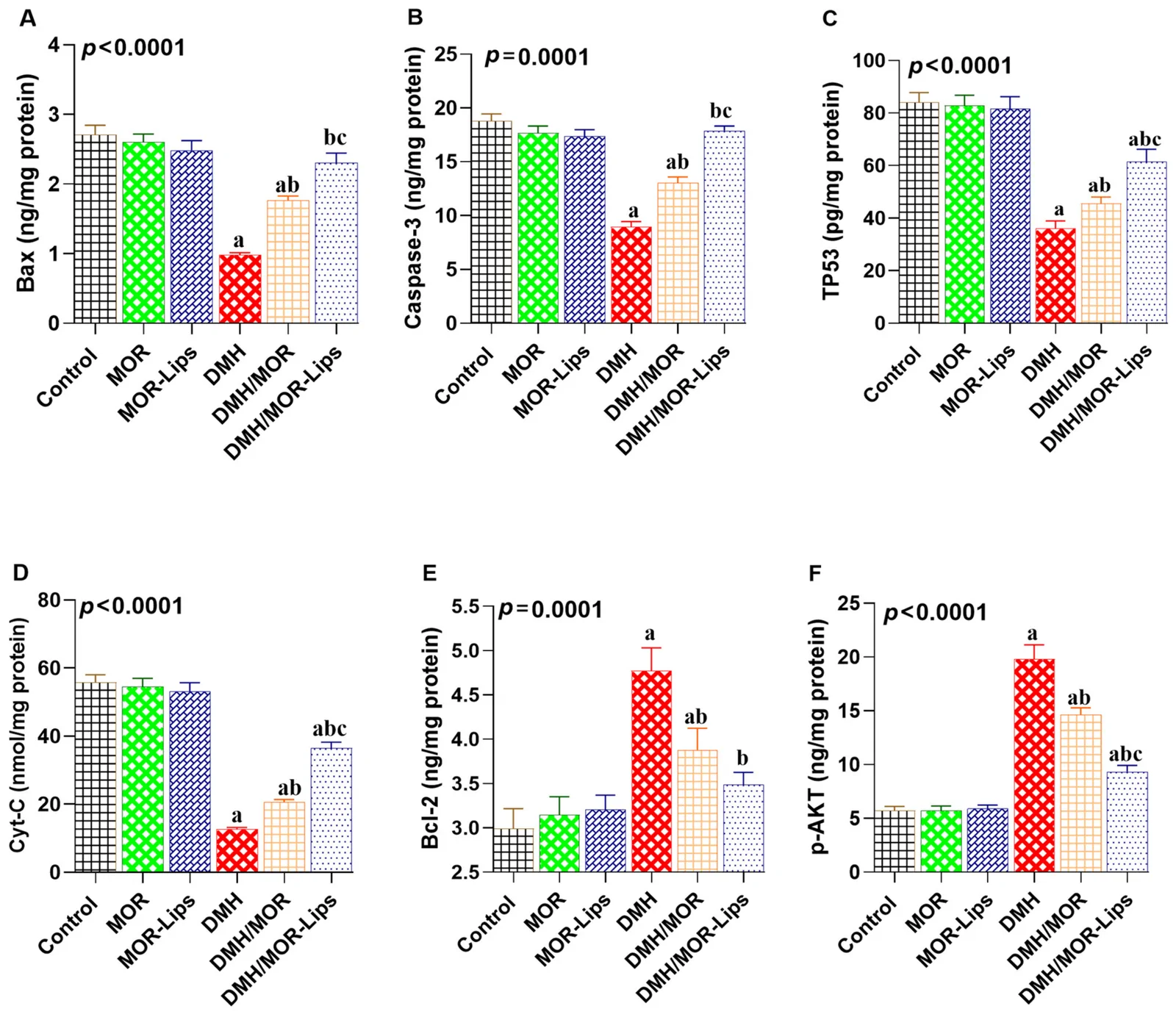
Effect of free and liposomal morin on apoptotic and anti-apoptotic markers in DMH-induced colon carcinogenesis. (**A**) Bax (Bcl-2-associated X protein), (**B**) Caspase-3, (**C**) p53 (Tumor Protein p53), (**D**) Cyt-C (Cytochrome c), (**E**) BCL-2 (B-cell lymphoma 2), (**F**) p-AKT (Phosphorylated Protein Kinase B). Mean ± SEM is used to present the results; annotations (a–c) indicate significant differences (*p* < 0.05) when compared with control (a), DMH (b), and crude MOR (c).

DMH significantly suppressed the pro-apoptotic markers Bax and caspase-3 and reduced p53 and cytochrome c, while concomitantly elevating the anti-apoptotic protein BCL-2 and the survival kinase p-AKT (Figure 8A–F). Free morin partially reversed these changes, whereas MOR-Lips produced a more pronounced pro-apoptotic shift. Bax, caspase-3, p53, and cytochrome c were restored toward or to control levels in the DMH + MOR-Lips group, and p-AKT was significantly reduced. BCL-2 was also lowered by both formulations, with a trend favoring MOR-Lips, although the difference between the two treatments was less marked than for p-AKT.

### 2.10. Effects on Autophagy-Related Markers

**Figure 9:**
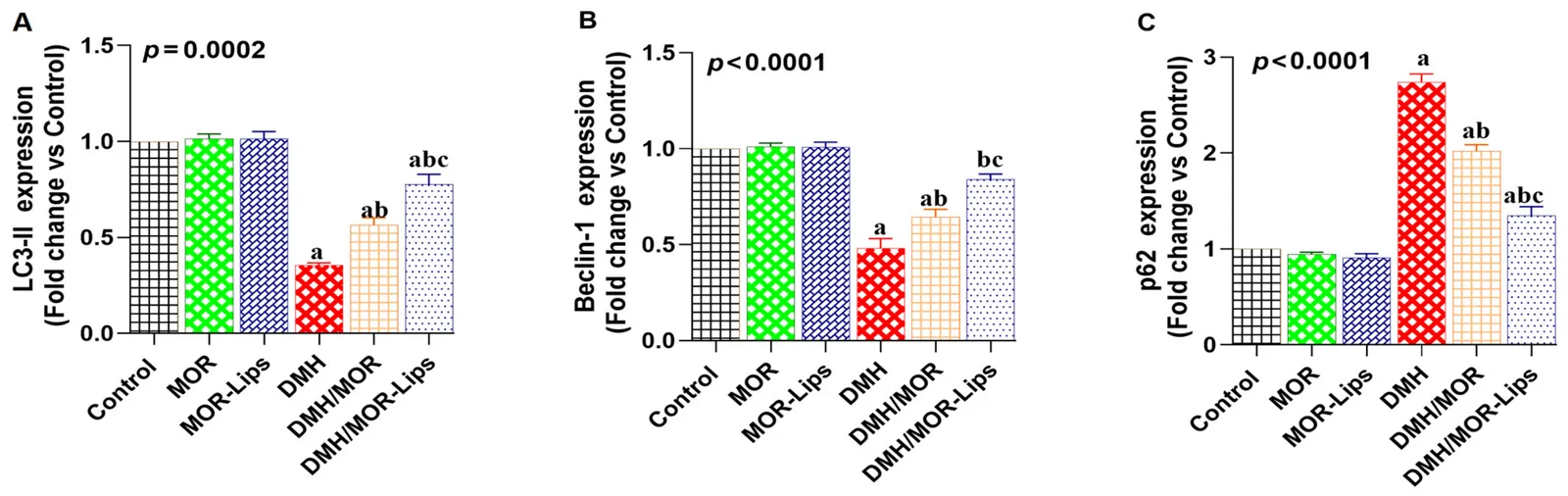
Effect of Crude and Liposomal Morin on Autophagy-Related Markers in DMH-Induced Colon Carcinogenesis. (**A**) LC3-II (Microtubule-associated protein 1A/1B-light chain 3 II), (**B**) Beclin-1, (**C**) P62 (Sequestosome 1). Mean ± SEM is used to present the results; annotations (a–c) indicate significant differences (*p* < 0.05) when compared with control (a), DMH (b), and crude MOR (c).

DMH exposure significantly downregulated LC3-II and Beclin-1 and upregulated p62, indicating impaired autophagic activity (Figure 9A–C). Free morin improved LC3-II and Beclin-1 expression and reduced p62, while MOR-Lips induced a stronger restoration of all three markers. Although LC3-II, Beclin-1, and p62 did not fully normalize in the DMH + MOR-Lips group, their levels were substantially closer to control values than in DMH alone, supporting partial reactivation of autophagy by the nano formulation.

### 2.11. Histopathological Changes in Colonic Tissue

**Figure 10:**
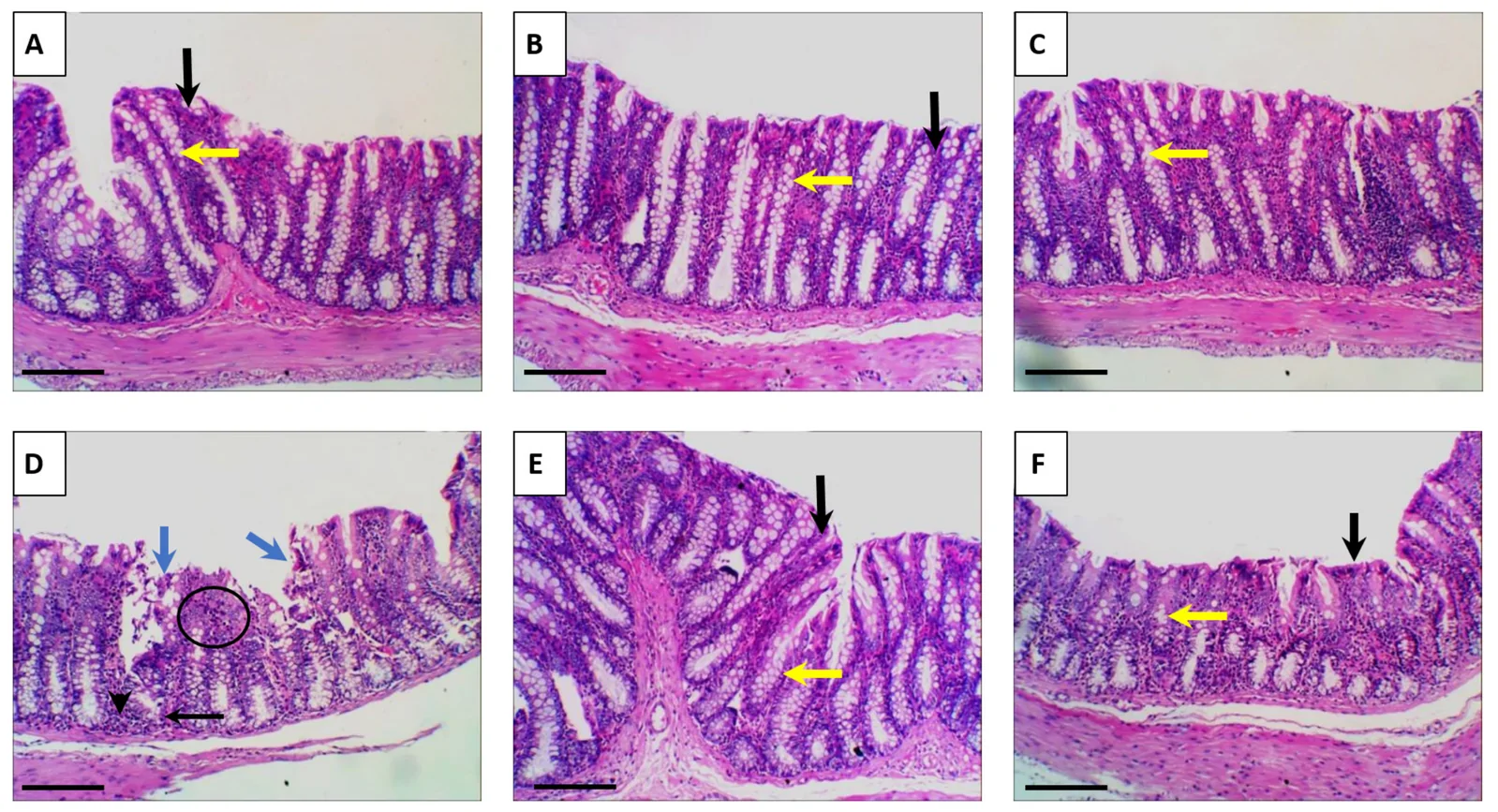
Representative photomicrographs of colonic tissue sections across experimental groups. (**A**) Control, (**B**) MOR, and (**C**) MOR-Lips showing normal histoarchitecture (black arrows), normal epithelial layer, and normal goblet cell localization (yellow arrows). (**D**) DMH group displaying severe crypt distortion (thin arrow), epithelial atypia (blue arrow) with hyperchromatic nuclei (circles), a marked loss (depletion) of goblet cells, and inflammatory cell infiltration (arrowhead). (**E**) DMH + MOR group showing moderate histological improvement with reduced inflammatory infiltrates. (**F**) DMH + MOR-Lips group exhibiting marked recovery with preserved crypt architecture and restored goblet cells. Scale bars = 100 µm (×200 magnification).

**Figure 11:**
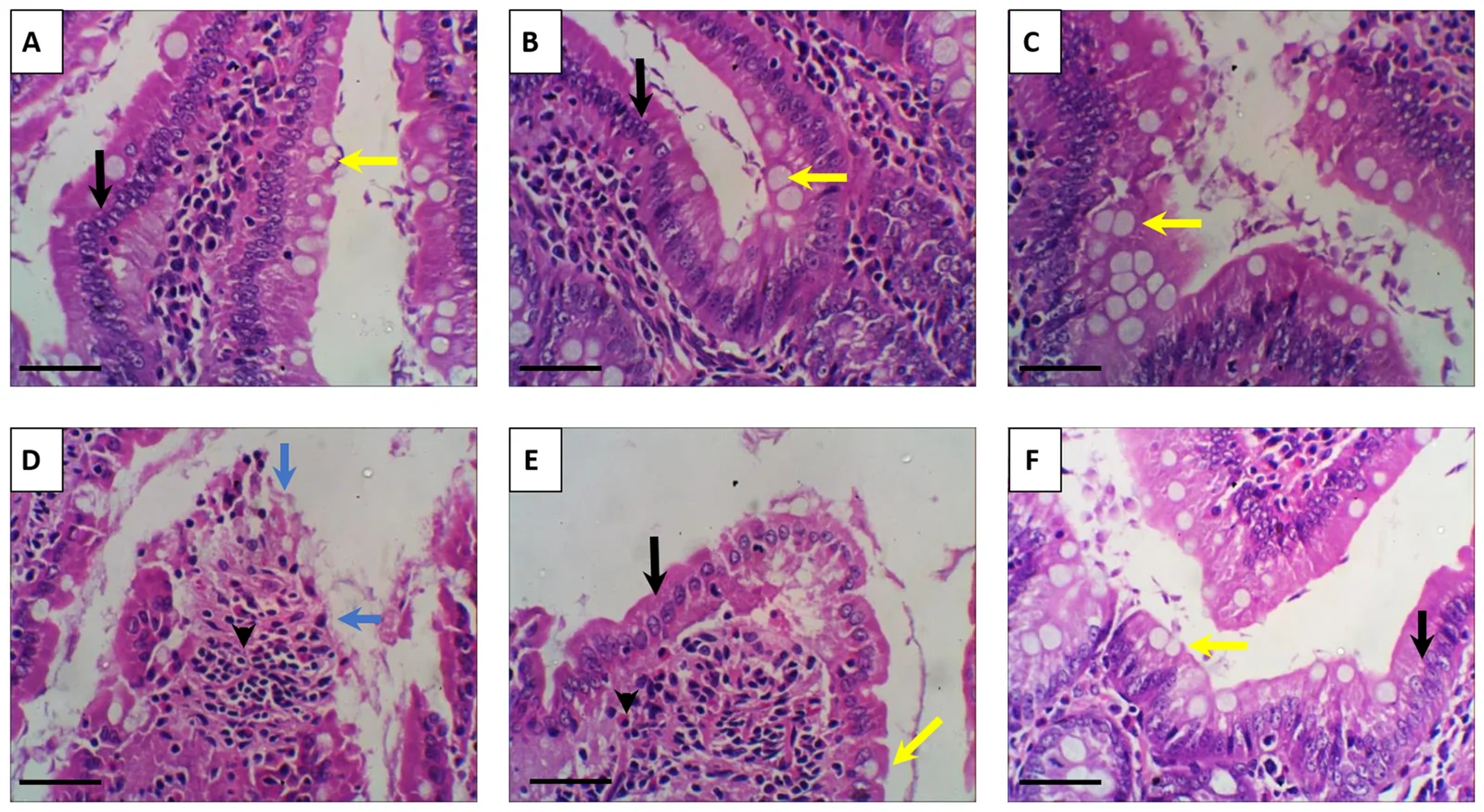
Representative high-power photomicrographs of colonic tissue sections across experimental groups focusing on the epithelial layer. (**A**) Control, (**B**) MOR, and (**C**) MOR-Lips show an intact, normal epithelial lining (black arrows) with regularly arranged colonocytes and normal goblet cell localization (yellow arrows). (**D**) DMH group displaying severe epithelial dysplasia and atypia (blue arrow) characterized by prominent hyperchromatic nuclei, marked depletion of mucin-producing goblet cells, and inflammatory cell infiltration (arrowhead). (**E**) DMH + MOR group showing moderate improvement in epithelial integrity with partially restored goblet cells and reduced inflammatory infiltrates (arrowhead). (**F**) DMH + MOR-LNPs group exhibiting marked epithelial recovery and regeneration, showing improvement with a highly preserved epithelial layer and repopulated goblet cells, closely resembling the normal control samples. Scale bars = 50 µm (×400 magnification).

Control, MOR, and MOR-Lips groups showed preserved colonic architecture, with orderly crypts, intact mucosa, and abundant goblet cells (Figure 10A–C and Figure 11A–C). In contrast, DMH-treated rats exhibited severe architectural distortion, crypt irregularity and fusion, epithelial atypia (loss of polarity, nuclear hyperchromasia), and dysplastic glands showing architectural distortion accompanied by dense inflammatory infiltrates (Figure 10D and Figure 11D). Co-treatment with free morin partially protected tissue structure, improving crypt alignment and reducing epithelial atypia and inflammation (Figure 10E and Figure 11E). The DMH + MOR-Lips group showed the most pronounced histopathological improvement, with near-normal crypt architecture, restoration of goblet cells, and minimal inflammatory infiltration (Figure 10F and Figure 11F).

### 2.12. NRF2 Immunohistochemical Expression

**Figure 12:**
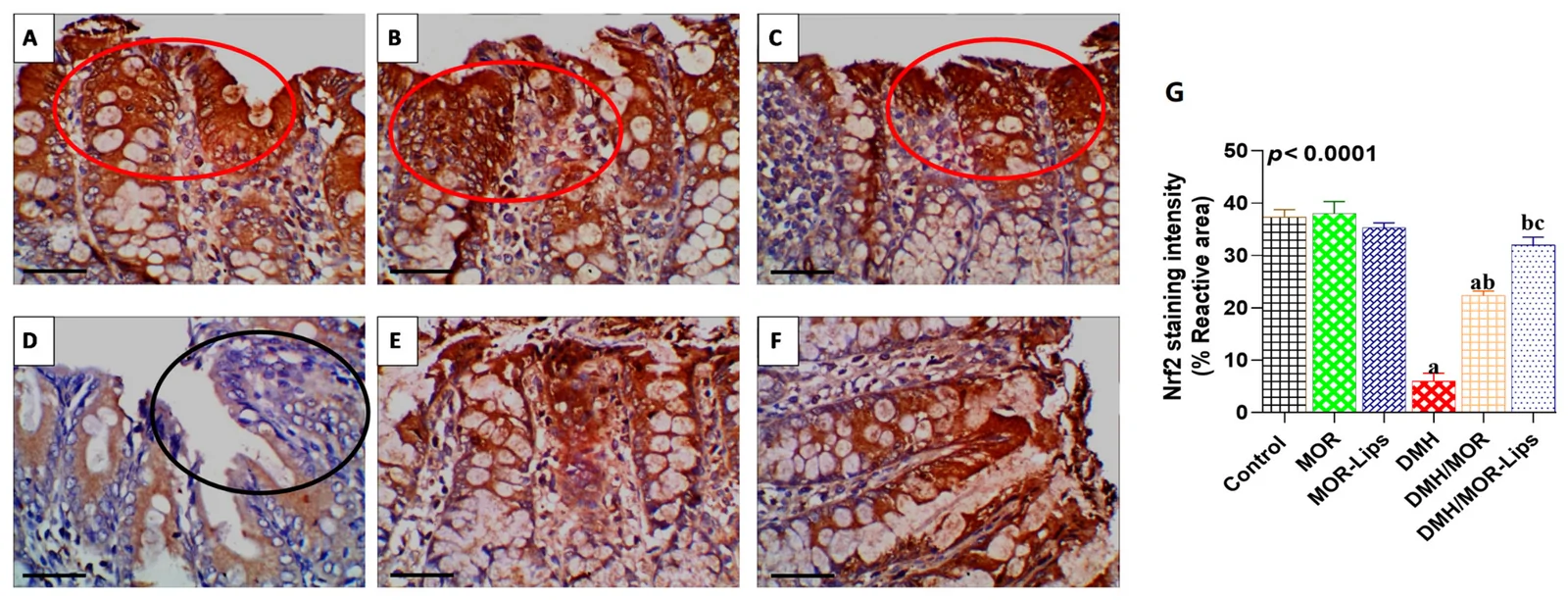
Effect of Crude and Liposomal Morin on NRF2 Immunoexpression in Colonic Tissue. Representative immunohistochemical photomicrographs of colon sections from the control and experimental groups: (**A**) Control; (**B**) MOR; (**C**) MOR-Lips; (**D**) DMH; (**E**) DMH + MOR; and (**F**) DMH + MOR-Lips. Red circles indicate strong positive NRF2 immunoreactivity within epithelial cells, whereas black circles denote negative NRF2 expression. All images were captured at ×400 magnification; scale bar = 50 µm. (**G**) Nrf2 immunohistochemical expression in colonic tissue. Mean ± SEM is used to present the results; annotations (a–c) indicate significant differences (*p* < 0.05) when compared with control (a), DMH (b), and crude MOR (c).

Immunohistochemistry revealed strong nuclear/cytoplasmic NRF2 expression in the control, MOR, and MOR-Lips groups (Figure 12A–C). DMH markedly reduced NRF2 immunoreactivity in colonic epithelial cells (Figure 12D), consistent with biochemical and molecular findings. Both treatments significantly increased NRF2 staining, with MOR-Lips showing a greater restorative effect than free morin (Figure 12E,F). Quantitative analysis confirmed a sharp decline in NRF2 immunoscore in the DMH group and a significant recovery in both treated groups, with values in the DMH + MOR-Lips group not significantly different from controls (Figure 12G).

### 2.13. NF-κB Immunohistochemical Expression

**Figure 13:**
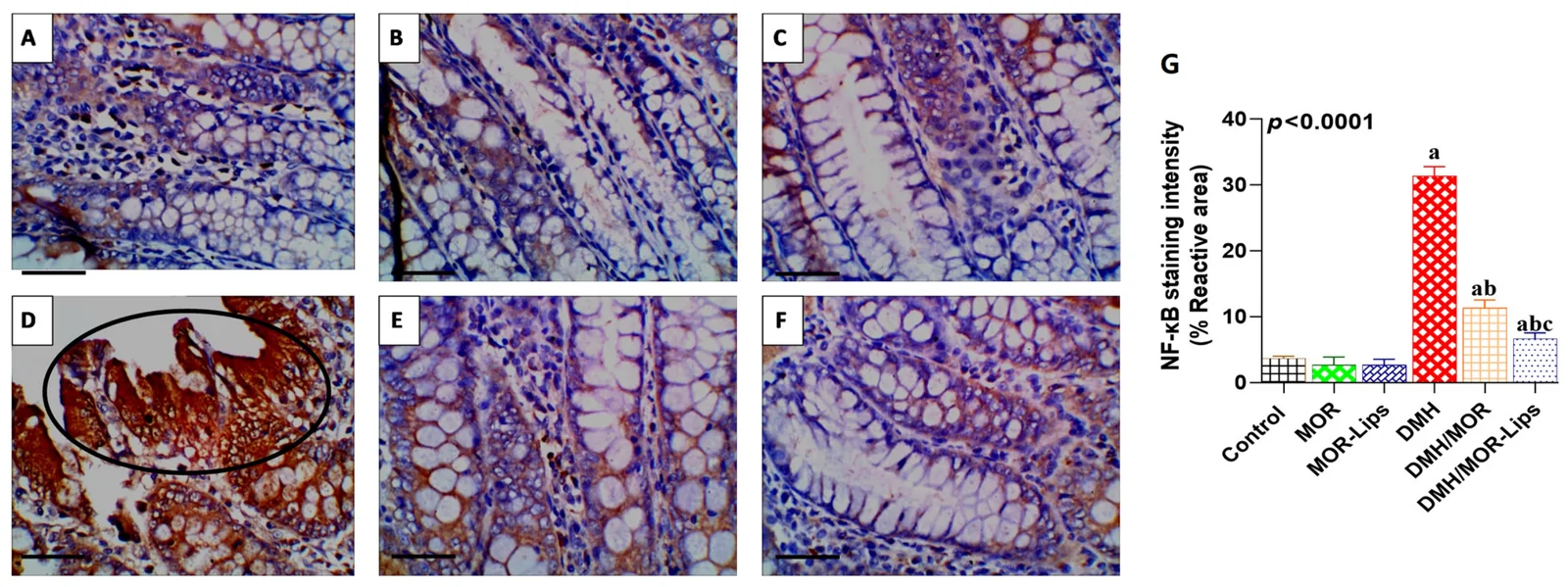
Effect of Crude and Liposomal Morin on NF-κB Immunoexpression in Colonic Tissue. Representative immunohistochemical photomicrographs of colon sections from the control and experimental groups: (**A**) Control; (**B**) MOR; (**C**) MOR-Lips; (**D**) DMH; (**E**) DMH + MOR; and (**F**) DMH + MOR-Lips. Representative immunohistochemical photomicrographs demonstrating NF-κB expression in colonic epithelial cells. Black circles highlight areas of strong positive immunoreactivity. All images were obtained at ×400 magnification with a scale bar of 50 µm. (**G**) NF-κB immunohistochemical expression in colonic tissue. Mean ± SEM is used to present the results; annotations (a–c) indicate significant differences (*p* < 0.05) when compared with control (a), DMH (b), and crude MOR (c).

In the control, MOR, and MOR-Lips groups, NF-κB expression was limited to low basal levels (Figure 13A–C). DMH administration markedly enhanced NF-κB immunoreactivity in the colonic epithelium, mainly as strong cytoplasmic staining (Figure 13D). Treatment with morin attenuated this DMH-induced overexpression, and MOR-Lips again produced a more pronounced reduction (Figure 13E,F). Quantitatively, NF-κB immunoscores were significantly elevated in the DMH group and significantly decreased in both treatment groups, with the DMH + MOR-Lips group reaching levels comparable to those of controls (Figure 13G).

## 3. Discussion

The present study comprehensively evaluated the protective activity of morin-loaded liposomes (MOR-Lips) against DMH-associated colonic alterations in a rat model of DMH-induced CRC. Multiple biochemical, molecular, and histological indicators related to tumor progression, metabolic reprogramming, oxidative and nitrosative stress, inflammation, proliferation, apoptosis, and autophagy were examined to characterize patterns associated with MOR-Lips treatment. The marked elevation of CEA, CA19-9, and CA125 in the DMH group was consistent with DMH-associated biochemical and histopathological alterations in this model [30,31]. However, because quantitative preneoplastic and neoplastic endpoints were not assessed, these biomarkers should not be interpreted as direct evidence of tumor incidence, tumor burden, or malignant transformation. Clinically, CEA correlates with tumor growth, metastatic potential, and epithelial abnormalities in CRC [32], while CA19-9 is frequently associated with tumor progression and aggressive disease behavior [33]. CA125 has also been linked to gastrointestinal cancer progression, particularly in advanced stages with peritoneal involvement [34]. The combined elevation of these markers in DMH-treated rats therefore appears to reflect a pattern consistent with DMH-associated pathological change.

In this context, both free morin and MOR-Lips were associated with lower CEA, CA19-9, and CA125 levels compared with DMH alone, with MOR-Lips showing a more pronounced effect. This pattern suggests that the liposomal formulation is associated with improved control of tumor-associated biomarker profiles and may confer greater interference with pathways involved in colorectal carcinogenesis. The observed findings are consistent with the reported antioxidant, anti-inflammatory, and antiproliferative properties of morin [17] and with the notion that liposomal encapsulation can enhance stability, solubility, bioavailability, and tissue penetration [27]. Consistently, Xie reported that morin administration in DMH-induced CRC coincided with reduced expression of glucose transporter member 1, hexokinase 2, and lactate dehydrogenase, and with attenuated tumor glucose utilization [9].

The increase in colonic HMG-CoA reductase levels observed in DMH-treated rats was in line with a shift towards enhanced mevalonate pathway activity [35]. As this pathway supplies isoprenoid intermediates and cholesterol, which are necessary for membrane synthesis, protein prenylation, and oncogenic signaling [36,37], its upregulation may be associated with metabolic reprogramming in colorectal cancer. In the present work, MOR-Lips showed a more pronounced reduction in HMG-CoA reductase levels than free morin. This pattern suggests that the liposomal formulation may modulate tumor-related metabolic pathways more effectively, in agreement with reports emphasizing cholesterol metabolism as a relevant feature and potential target in colorectal cancer [36,38].

Consistent with previous DMH studies, DMH exposure in this model was associated with marked oxidative and nitrosative stress. The elevation of MDA, NO, and 8-OHdG coincided with biochemical evidence of lipid peroxidation, nitrosative injury, and oxidative DNA damage [5,8,22,39,40,41,42]. Simultaneously, lower levels of GSH and reduced activities of SOD and CAT were consistent with compromised antioxidant defenses [42,43,44]. These changes were accompanied by downregulated NRF2 and HO-1 expression, suggesting impairment of a key cytoprotective axis. Because NRF2 controls multiple antioxidant and phase II detoxifying genes [45,46], and reduced NRF2/HO-1 signaling has been associated with greater susceptibility to carcinogen-induced oxidative stress [47,48], the present pattern points to an NRF2/HO-1-deficient redox state in DMH-exposed colonic tissue.

In contrast, MOR-Lips co-treatment was associated with lower MDA, NO, and 8-OHdG and with partial or near-complete normalization of GSH, SOD, CAT, NRF2, and HO-1 compared with DMH alone, and these changes were generally more pronounced than those observed with free morin. This profile suggests that the liposomal formulation is linked to a more favorable redox balance and a more robust NRF2/HO-1 response. Previous studies have reported that morin can activate NRF2 signaling and upregulate antioxidant genes, thereby diminishing oxidative damage [10,49]. In line with these reports, Xie described that morin administration in DMH-induced CRC was associated with reduced oxidative stress and lower tumor burden [9].

Inflammatory changes observed in the DMH group, namely increased TLR4, NF-κB, and COX-2, together with elevated TNF-α, IL-6, IL-1β, and MPO, were consistent with activation of a pro-tumorigenic inflammatory axis [5]. TLR4 is a key pattern recognition receptor whose engagement has been associated with NF-κB activation and pro-inflammatory gene expression [11]. NF-κB, in turn, has been linked to inflammation-driven carcinogenesis and regulation of cytokines, chemokines, adhesion molecules, and anti-apoptotic factors [13,50]. COX-2 upregulation has been associated with increased PGE2 production and with pro-proliferative, pro-angiogenic, and anti-apoptotic conditions in the tumor microenvironment [51]. Elevated MPO activity is typically interpreted as a marker of enhanced neutrophil infiltration and generation of reactive oxidants [12]. In the present study, both morin preparations were associated with lower levels of these inflammatory mediators relative to DMH alone, with MOR-Lips consistently showing greater attenuation, particularly for TLR4, NF-κB mRNA, COX-2, TNF-α, IL-1β, and MPO. The NF-κB and COX-2 patterns observed here are consistent with earlier reports linking morin administration to modulation of NF-κB signaling and inflammatory mediators in colorectal cancer models [50].

Regarding proliferation, the increases in Ki-67 and PCNA observed in the DMH group agreed with enhanced proliferative activity [14,15]. Co-treatment with morin formulations was associated with lower Ki-67 and PCNA, and MOR-Lips were linked to a more pronounced decrease, with Ki-67 returning to control-comparable levels. These findings are compatible with an association between MOR-LIP treatment and dampened proliferative signaling. Mechanistically, prior work has associated morin with p53 activation and suppression of NF-κB and AKT pathways in colorectal cancer models [9,52], and with downregulation of CD133 and PUM1 in colorectal cancer stem-like cells [26]. Although these mechanisms were not directly interrogated at the functional level in our study, the patterns observed in p53 and p-AKT are consistent with such associations.

The apoptotic profile observed in DMH-treated rats (lower Bax, caspase-3, p53, and cytochrome c and higher BCL-2 and p-AKT) was consistent with an anti-apoptotic, survival-oriented phenotype. Reduced p53 is commonly associated with impaired DNA damage responses and persistence of mutated clones [53], while elevated AKT has been linked to enhanced survival and proliferation signals [16]. In contrast, MOR-Lips co-treatment coincided with higher Bax, caspase-3, p53, and cytochrome c, and lower BCL-2 and p-AKT compared with DMH alone, with changes more pronounced than those associated with free morin. These patterns are compatible with a shift toward re-engagement of intrinsic mitochondrial apoptosis. Earlier in vitro work has associated morin with inhibition of AKT phosphorylation, mitochondrial depolarization, increased ROS, and cytochrome c release in colorectal cancer cells [9,17], and the present in vivo data are in line with these observations at the biomarker level.

Autophagy-related changes in DMH-treated rats, reduced LC3-II and Beclin-1 with increased p62, were compatible with altered autophagy-related signaling and defective cellular clearance [54,55,56]. Such a profile has been associated with accumulation of damaged organelles, increased oxidative stress, and promotion of tumor progression. MOR-Lips co-treatment coincided with higher LC3-II and Beclin-1, and lower p62, compared with DMH alone, again more prominently than with free morin. These changes suggest that liposomal formulation is associated with partial restoration of autophagic activity. Recent reports have linked morin to modulation of autophagy in colorectal cancer, possibly via effects on the PI3K/AKT/mTOR axis and on autophagic flux [53,54], and the present data are consistent with this interpretation at the level of autophagy markers.

Histopathological and immunohistochemical findings further supported these biochemical and molecular patterns. DMH exposure was associated with severe distortion of colonic architecture, crypt irregularity and fusion, epithelial atypia, goblet cell depletion, and dense inflammatory infiltrates, together with reduced NRF2 and increased NF-κB immunoreactivity. Morin treatment coincided with partial improvement of these features, whereas MOR-Lips were associated with near-normal crypt organization, restoration of goblet cells, reduced inflammatory infiltrates, and NRF2 and NF-κB immunoscores approaching those of controls.

The pathways evaluated in the present study are biologically interconnected rather than independent. DMH-associated ROS/RNS generation may contribute to both suppression of the NRF2/HO-1 antioxidant response and activation of the TLR4/NF-κB/COX-2 inflammatory axis. Reduced NRF2/HO-1 activity may impair cellular antioxidant capacity, thereby permitting persistence of oxidative damage and amplifying inflammatory signaling [57]. In turn, sustained TLR4/NF-κB/COX-2 activation can promote cytokine production and survival signaling, thereby increasing proliferation and resistance to apoptosis by modulating BCL-2 family proteins, caspase activity, and AKT-related pathways [58]. Autophagy is also closely linked to redox regulation and inflammation: p62 acts as a regulatory interface between autophagy and NRF2 through interaction with KEAP1, whereas altered autophagy may influence cellular oxidative stress, inflammatory signaling, and cell survival [59]. Thus, the concurrent restoration of NRF2/HO-1 and autophagy-related markers, attenuation of TLR4/NF-κB/COX-2 signaling, and a shift toward a pro-apoptotic profile in MOR-LIP-treated rats are compatible with coordinated modulation of an oxidative stress–inflammation–survival network [19]. However, these mechanistic links were not directly tested in the present study and should be interpreted as biologically plausible associations rather than proof of causality.

**Figure 14:**
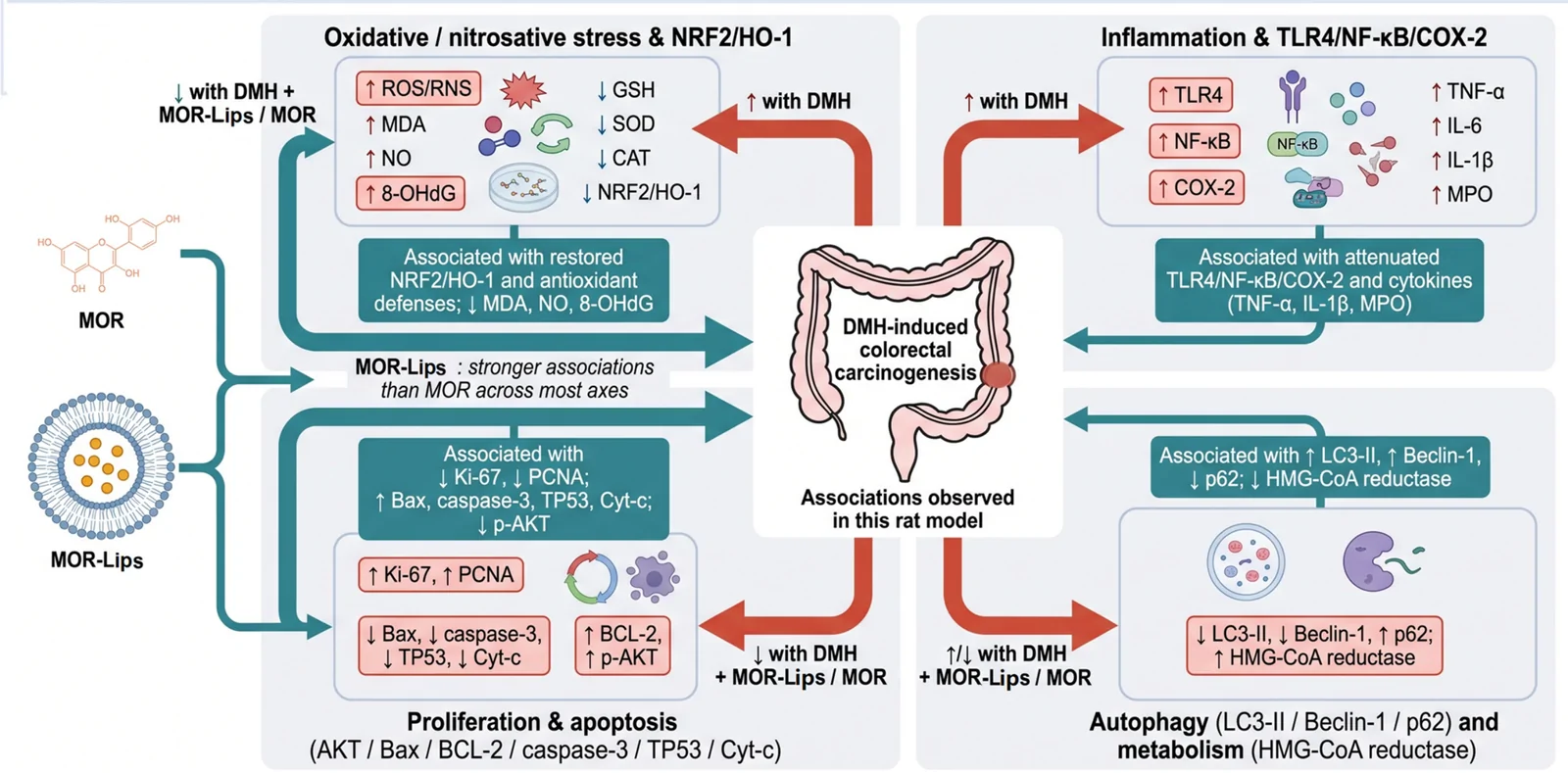
Proposed mechanistic model summarizing pathways associated with MOR and MOR-Lips in DMH-induced colorectal carcinogenesis. DMH exposure is associated with metabolic activation and increased production of reactive oxygen and nitrogen species (ROS/RNS), coinciding with downregulation of NRF2/HO-1 signaling, depletion of GSH, SOD, and CAT, and elevated levels of MDA, NO, and 8-OHdG. These changes are associated with enhanced TLR4/NF-κB/COX-2 signaling and increased levels of TNF-α, IL-6, IL-1β, and MPO, contributing to a pro-inflammatory tumor microenvironment. In parallel, DMH is associated with higher HMG-CoA reductase activity and mevalonate pathway engagement, increased proliferative markers (Ki-67, PCNA), reduced pro-apoptotic proteins (Bax, caspase-3, p53, cytochrome c), increased anti-apoptotic and survival mediators (BCL-2, p-AKT), and altered autophagy-related markers (decreased LC3-II and Beclin-1 with increased p62), compatible with altered autophagy-related signaling. Co-treatment with morin, and more prominently with morin-loaded liposomes (MOR-Lips), is associated with restoration of NRF2/HO-1 and antioxidant defenses, attenuation of oxidative and inflammatory markers, partial normalization of HMG-CoA reductase, reduced Ki-67 and PCNA, a shift towards a pro-apoptotic profile, and partial recovery of autophagy-related markers. Collectively, MOR-Lips are associated with a multi-pathway pattern that resembles a less tumor-promoting phenotype in this DMH model.

Taken together, the data indicate that MOR-Lips were consistently associated with more favorable metabolic, redox, inflammatory, proliferative, apoptotic, and autophagy-related marker profiles than free morin in this DMH model. The proposed integrated, but non-causal, mechanistic framework is summarized in Figure 14.

Several limitations of this study should be considered when interpreting these findings. First, all experiments were conducted in a single chemical carcinogenesis model (DMH-induced CRC in rats), which, although widely used and relevant, does not fully capture the genetic heterogeneity and microenvironmental complexity of human colorectal cancer. In addition, quantitative preneoplastic and neoplastic endpoints, including aberrant crypt foci, tumor incidence, tumor multiplicity, and tumor burden, were not assessed. Therefore, although the study documented DMH-associated biochemical, molecular, and histopathological alterations, it cannot establish whether MOR or MOR-Lips directly prevent, delay, or reduce colorectal neoplastic lesion development. Second, pharmacokinetic, biodistribution, and colon-targeting properties of MOR-Lips were not directly characterized; therefore, the extent to which the observed associations are driven by altered systemic exposure or tissue accumulation remains to be determined. Third, only one morin dose and one liposomal composition were examined, limiting conclusions regarding dose–response relationships and formulation optimization. Fourth, long-term safety and potential interactions with standard chemotherapeutic agents were not investigated. Moreover, hematological parameters were not assessed; therefore, potential systemic effects of MOR and MOR-Lips on hematological indices could not be determined. Comprehensive safety assessment and herb–drug interaction studies were beyond the scope of the present work. This issue may be relevant in patients receiving urate-lowering therapy, particularly febuxostat or allopurinol. In a rat pharmacokinetic study, morin pretreatment increased febuxostat exposure, including C_max_, the area under the concentration–time curve, and the elimination half-life, possibly through inhibition of CYP-mediated metabolism and/or P-gp-mediated efflux [60]. Although these findings cannot be directly extrapolated to MOR-Lips or humans, they suggest the potential for altered febuxostat disposition when morin is coadministered. Direct evidence regarding interactions between morin or MOR-Lips and allopurinol remains insufficient. Therefore, co-administration with either febuxostat or allopurinol should be approached cautiously until dedicated pharmacokinetic and safety studies are performed. Such studies should evaluate circulating drug and metabolite concentrations, serum urate levels, liver and kidney function, hematological indices, and histopathological endpoints of toxicity. Western blot analysis of apoptotic and anti-apoptotic markers was also not performed, and future studies should include such validation to further substantiate the observed apoptosis-related molecular changes. Importantly, because 30% ethanol was used as the vehicle, the control group represented a vehicle control rather than a completely untreated control. An empty-liposome group was also not included. Consequently, potential contributions of ethanol or the liposomal carrier to Nrf2 signaling, oxidative stress, and mucosal integrity cannot be completely excluded. The stability of MOR-Lips in the 30% ethanol administration vehicle was not directly assessed. Future studies should evaluate particle size, PDI, and morin retention under actual dosing conditions. Additionally, future studies incorporating both an untreated control and an empty liposome group would help distinguish the effects attributable to MOR and MOR-Lips from those attributable to the vehicle or liposomal carrier. Finally, causal involvement of specific pathways (e.g., NRF2/HO-1, TLR4/NF-κB/COX-2, AKT, or autophagy regulators) was not formally tested using genetic or pharmacological modulation; therefore, the relationships reported here should be interpreted as associative rather than definitively causal.

From a translational perspective, the present findings should be considered hypothesis-generating and cannot establish the safety, optimal dose, pharmacokinetics, colonic exposure, or chemopreventive efficacy of MOR-Lips in humans. Before clinical evaluation, additional studies should assess pharmacokinetics, biodistribution, colon-targeting capacity, dose–response relationships, repeated-dose toxicity, and potential interactions with standard CRC therapies. Validation in independent animal cohorts (both sexes) and complementary CRC models, such as inflammation-associated, genetically engineered, orthotopic, or patient-derived models, where feasible, would help confirm reproducibility and external validity. If these preclinical requirements are satisfactorily addressed, initial clinical studies should focus on safety, tolerability, pharmacokinetics, and dose selection. Subsequent randomized trials should be adequately powered on a prespecified clinically meaningful endpoint rather than biomarker changes alone.

## 4. Materials and Methods

### 4.1. Preparation and Characterization of Morin-Loaded Liposomes (MOR-Lips)

**Figure 15:**
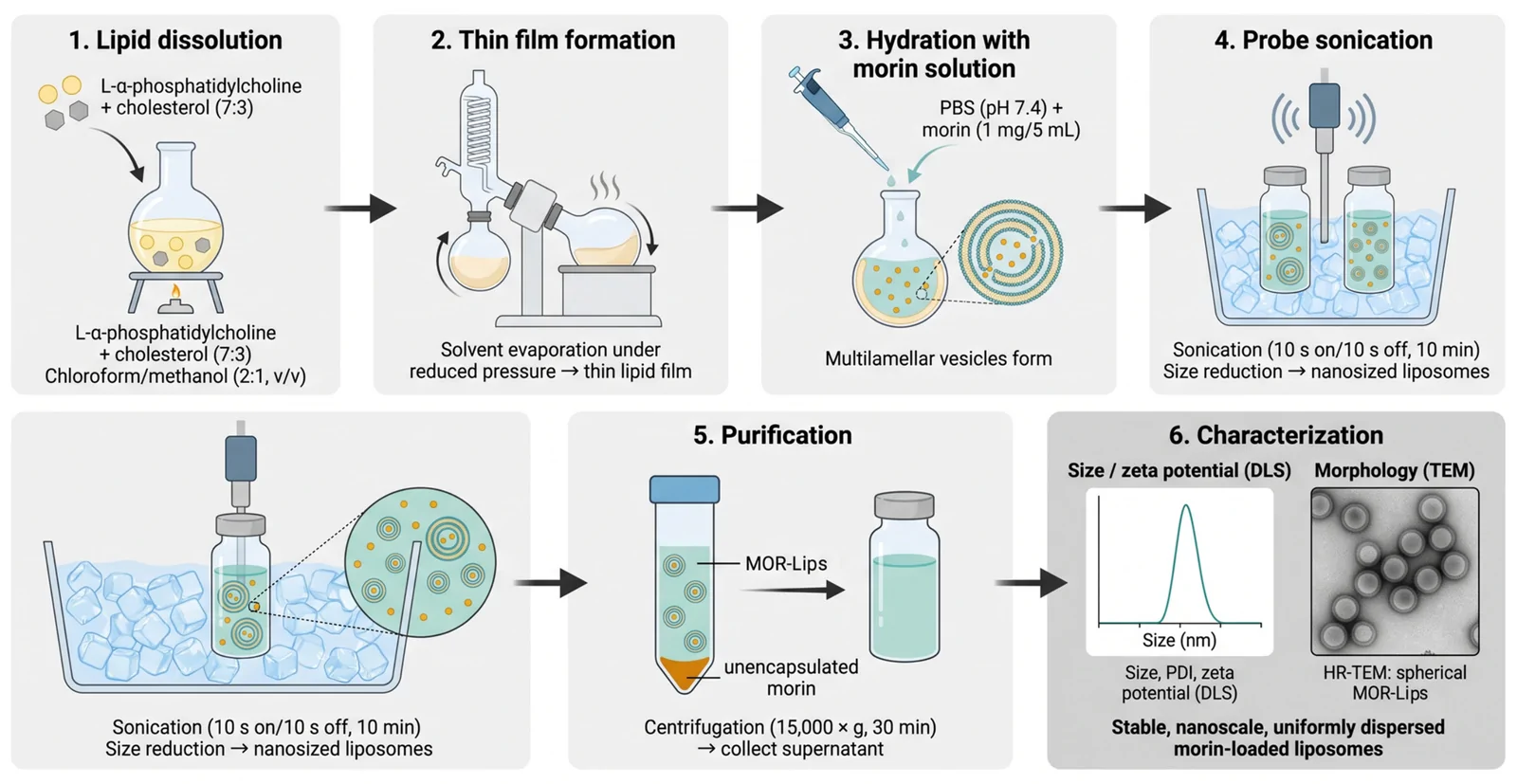
Schematic preparation and characterization of morin-loaded liposomes (MOR-Lips). It was generated with the assistance of “GAAbstract” (https://gaabstract.com/, accessed on 29 June 2026).

Morin was commercially purchased from Sigma-Aldrich (St. Louis, MO, USA) with a reported purity of ≥98%, as specified in the manufacturer’s Certificate of Analysis (CoA). The identity and purity of morin were confirmed based on the manufacturer’s quality control documentation and the CoA accompanying the supplied material. Morin-loaded liposomes (MOR-Lips) were prepared by a modified thin-film hydration method [61] as depicted in Figure 15. The resulting liposomal dispersion was sonicated using a Vibra-Cell™ probe sonicator (SONICS, Newtown, CT, USA). Particle size, polydispersity index (PDI), and zeta potential were determined by dynamic light scattering using a Zetasizer Nano ZS instrument (ZEN3600; Malvern Panalytical, formerly Malvern Instruments, Malvern, UK). Morphology was examined by high-resolution transmission electron microscopy (HR-TEM; JEOL JEM-2100, Tokyo, Japan).

### 4.2. Encapsulation Efficiency and Drug Loading of MOR

Encapsulation efficiency (EE, %) and drug loading (DL, %) of MOR-Lips were determined after separation of free (unencapsulated) morin by centrifugation at 15,000× g for 30 min. The supernatant containing free MOR was collected and quantified using a validated analytical method. The encapsulated drug content was calculated by subtracting free MOR from the total amount initially added (1 mg). Encapsulation efficiency was calculated as:

(1) $$EE\;\left(\%\right)=\frac{\left(Total\;MOR\;-Free\;MOR\right)}{Total\;MOR}\;\times 100$$

Drug loading was calculated relative to the total lipid content (100 mg) using:

(2) $$DL\;\left(\%\right)=\frac{\left(Entrapped\;MOR\right)}{\left(Total\;lipids\;+\;Entrapped\;MOR\right)}\;\times 100$$

All measurements were performed in triplicate.

### 4.3. In Vitro Release Study of MOR-Lips

The release behavior of morin from the liposomal formulation was examined using a dialysis membrane–based diffusion setup. An accurately measured volume of the MOR-Lip dispersion, corresponding to a defined morin dose, was transferred into dialysis tubing (molecular weight cut-off 12–14 kDa), which was then securely closed. The dialysis bag was placed in 50 mL of phosphate-buffered saline (PBS; P5119, pH 7.4, Sigma-Aldrich, St. Louis, MO, USA) supplemented with 0.5% Tween 80 to ensure sink conditions, and the mixture was maintained at 37 °C with gentle agitation. At scheduled sampling times, portions of the external medium were collected and immediately replaced with an equal volume of freshly pre-equilibrated buffer to preserve constant volume and composition. Morin concentrations in the sampled medium were determined using the established analytical assay, and each time point was measured in triplicate. The cumulative percentage of morin released from MOR-Lips was subsequently calculated and plotted as a function of incubation time.

### 4.4. FTIR Analysis of MOR-Lips

FTIR spectroscopy (PerkinElmer, Spectrum Two, Waltham, MA, USA) was used to assess possible interactions between MOR and the lipid components of the liposomal formulation. Pure MOR, blank liposomes, and MOR-Lips were analyzed separately. Each sample was mixed with potassium bromide (KBr; 221864 (FT-IR grade, ≥99%), Sigma-Aldrich, St. Louis, MO, USA), compressed into a transparent pellet, and analyzed. Spectra were recorded over the range of 4000–400 cm^−1^ at a spectral resolution of 4 cm^−1^, with 32 scans accumulated for each sample. Background spectra were collected under identical conditions before sample analysis and subtracted from the corresponding sample spectra. The resulting spectra were evaluated to identify characteristic functional groups and assess potential interactions among MOR and the formulation components.

### 4.5. Stability Study of MOR-Lips

The stability of MOR-Lips was evaluated during storage at 4 °C for 3 months. Samples were analyzed at 0, 1, 2, and 3 months. At each time point, particle size, PDI, and zeta potential were measured by dynamic light scattering. Morin retention was also determined to assess the stability of the encapsulated morin during storage. The stability assessment was performed using the stored MOR-Lips dispersion, without addition of the 30% ethanol administration vehicle. All measurements were performed in triplicate.

### 4.6. Animal Care, Acclimatization, and Environmental Conditions

Sixty healthy male Wistar rats (220–250 g) were obtained from the “MERC Center, Faculty of Medicine, Mansoura University, Egypt.” Animals were housed in clean, well-ventilated cages under standard laboratory conditions, with relative humidity of 50 ± 6.19%, temperature of 25 ± 2.13 °C, and a 12 h light/dark cycle. Rats were allowed to acclimatize for two weeks before the experiment. Throughout acclimatization and treatment, animals had free access to water and a standard pellet diet. Health status was monitored regularly.

The standard laboratory chow (Laboratory Rodent Diet 5001, LabDiet, St. Louis, MO, USA) contained 23% protein, 4.5% fat, 56% carbohydrates, 2.5% minerals, 6% fiber, and 8% ash, with an energy content of 3.36 kcal/g. All procedures complied with the OECD Guidelines for the Care and Use of Rodents and adhered to institutional ethical and standard research practices [62]. The study was approved by the Animal Care and Use Committee of the Faculty of Veterinary Medicine, Mansoura University, Egypt (approval code MU-ACUC; VM.R.26.07.310).

### 4.7. In Vivo Experimental Design and Treatment Protocols

Rats were randomly assigned to six groups (*n* = 10 per group). The group size was selected based on prior comparable DMH-induced colorectal-carcinogenesis studies [42,63,64] and practical/ethical considerations; no formal a priori power calculation was performed. Randomization was performed using a simple computer-generated random number list prepared in advance, and animals were allocated to the six experimental groups (control, MOR, MOR-Lips, DMH, DMH + MOR, DMH + MOR-Lips) based on this sequence.

- Group 1 (vehicle control) received 30% ethanol (vehicle) by oral gavage once daily for 10 weeks. Thus, this group served as a vehicle control rather than a completely untreated control.
- Group 2 received free MOR (100 mg/kg body weight), dissolved in 30% ethanol, once daily for 10 weeks. This dose was selected based on previously published rodent studies reporting biological activity and tolerability of MOR at this dose [52,65,66].
- Group 3 received MOR-Lips containing MOR at an equivalent dose of 100 mg/kg body weight once daily for 10 weeks.
- Group 4 (DMH) received dimethylhydrazine (DMH; 20 mg/kg body weight in 0.9% saline) once weekly for 10 weeks, as described previously [67], together with daily 30% ethanol as vehicle.
- Group 5 received DMH as in Group 4 plus free MOR (100 mg/kg body weight) daily for 10 weeks.
- Group 6 received DMH as in Group 4 plus MOR-Lips containing MOR at an equivalent dose of 100 mg/kg body weight once daily for 10 weeks.

All treatments were freshly prepared before administration. The liposomal formulation was suspended in 30% ethanol to maintain dosing consistency. No completely untreated control group or empty-liposome group was included in the experimental design. Body weight was recorded weekly to adjust DMH dosing accurately.

To minimize potential confounders, animals from different groups were housed in identical cages under the same environmental conditions described in Section 4.5, and handling and dosing were performed at similar times of day throughout the study. The order of treatments and sample collection followed a rotating schedule across groups to avoid systematic bias.

### 4.8. Tissue Handling and Processing

To limit inter-animal variability at sacrifice, rats were fasted for approximately 10 h before sampling. Anesthesia was induced with isoflurane, and the absence of withdrawal reflexes confirmed the depth of anesthesia. Euthanasia was then completed by continued exposure to 5% isoflurane in oxygen for 5 min, and the absence of cardiac activity and spontaneous respiration confirmed death.

For subsequent analyses, seven animals per group were designated for biochemical and molecular measurements, and three animals per group were reserved for histological examination. Whole blood was collected into plain tubes, allowed to clot at room temperature, and centrifuged at 3000 rpm for 10 min at 4 °C. The resulting serum was separated carefully to avoid hemolysis, aliquoted, and stored at −80 °C until use.

Immediately after euthanasia, colonic segments were dissected, gently rinsed with ice-cold normal saline to remove luminal contents and residual blood, and processed without delay. Each colon was divided into two parts: one portion was blotted dry, wrapped in aluminum foil, and frozen at −80 °C for biochemical assays; the other portion was fixed in 10% neutral buffered formalin for histopathological, ultrastructural, and molecular evaluations. For tissue homogenates, colon samples were suspended in cold phosphate buffer (50 mM, pH 7.4) to yield 10% (*w*/*v*) homogenates. The homogenates were centrifuged at 2600× *g* for 20 min to remove particulate material, and the clarified supernatant was collected and stored at −80 °C for subsequent biochemical and molecular analyses.

### 4.9. Serum Biochemical and Tumor Marker Analysis

Serum carcinoembryonic antigen (CEA), carbohydrate antigen 19-9 (CA19-9), and carbohydrate antigen 125 (CA125) were quantified using ELISA kits according to the manufacturers’ instructions. CEA was measured using a rat sandwich ELISA kit (Elabscience Biotechnology Co., Ltd., Wuhan, China, Cat. No. E-EL-R0150) based on a pre-coated 96-well plate and a biotin–avidin HRP system. After incubation and washing, color development was measured at 450 nm, and concentrations were calculated from a standard curve (detection range 0.16–10 ng/mL). CA19-9 was determined using a QuickTest ELISA kit (Fine Biotech Co., Ltd., Wuhan, China; Cat. No. ER1907) with a detection range of 0.625–40 IU/mL and sensitivity of 0.375 IU/mL. CA125 was measured using a competitive ELISA kit (Cat. No. MBS732014, MyBioSource, San Diego, CA, USA), with a detection range of 5.0–100 U/mL and sensitivity of 1.0 U/mL.

### 4.10. Assessment of Oxidative Stress and Antioxidant Defense Markers

Oxidative stress and antioxidant parameters in colon tissue were measured using commercial kits from Bio-Diagnostic Co., Ltd. Cairo, Egypt, following the manufacturer’s instructions. Lipid peroxidation was assessed by quantifying malondialdehyde (MDA) using the TBARS assay (Cat. No. MD 25 29). MDA forms a pink chromogen with thiobarbituric acid at 95 °C in an acidic medium, measured at 534 nm and expressed as nmol/g tissue. Nitric oxide (NO) was determined using the Griess colorimetric assay (Cat. No. NO 25 33), which measures nitrite (NO_2_^−^) at 540 nm; values were expressed as µmol/g tissue.

Superoxide dismutase (SOD) activity was measured using a colorimetric inhibition assay (Cat. No. SD 25 21) based on inhibition of nitroblue tetrazolium reduction in the presence of phenazine methosulfate, with absorbance at 560 nm and results expressed as U/mg protein. Catalase (CAT) activity was measured using a colorimetric kit (Cat. No. CA 25 17) that quantifies the decomposition of H_2_O_2_; unreacted H_2_O_2_ forms a colored complex, and CAT activity is expressed as U/mg protein. Reduced glutathione (GSH) was measured using a colorimetric kit (Cat. No. GR 25 11) based on the reaction with DTNB to form a yellow chromogen, read at 405 nm and expressed as mmol/g tissue.

Levels of 8-hydroxy-2′-deoxyguanosine (8-OHdG) were determined using a rat ELISA kit (MyBioSource, Cat. No. MBS165204). Samples and standards were added to anti-8-OHdG pre-coated plates along with biotinylated antibody and Streptavidin-HRP. After incubation, washing, and substrate addition, absorbance was measured at 450 nm, and concentrations were calculated from the standard curve.

Nuclear factor erythroid 2–related factor 2 (NRF2) and heme oxygenase-1 (HO-1) levels in colon tissue were measured using MyBioSource ELISA kits (Cat. Nos. MBS3807961 and MBS2024438, respectively). Absorbance was read at 450 nm, and all measurements were performed in triplicate and expressed as ng/mg protein.

### 4.11. Evaluation of Major Inflammatory Mediators

Colonic inflammatory mediators were quantified using rat-specific ELISA kits (MyBioSource, San Diego, CA, USA; Assay Genie). TLR4, NF-κB, and COX-2 were measured using MyBioSource kits (Cat. Nos. MBS161614, MBS287521, and MBS266603, respectively). TNF-α, IL-6, and IL-1β were determined using Assay Genie kits (Cat. Nos. AEES00516, RTES00015, and AEES02613, respectively). Assays were performed strictly according to the manufacturers’ protocols. Total protein content was used for normalization, and absorbance was read at 450 nm. All samples were analyzed in triplicate. Each plate included blank and quality control wells to verify performance and correct for background.

Colonic myeloperoxidase (MPO) activity was measured spectrophotometrically as described by Haqqani et al. [68]. The assay is based on MPO-catalyzed oxidation of o-dianisidine in the presence of H_2_O_2_, yielding a colored product measured at 460 nm. Enzyme activity was calculated using a molar extinction coefficient of 1.3 × 10^4^ M^−1^·cm^−1^ and expressed as units, where one unit represents the amount of enzyme that degrades 1 μmol H_2_O_2_ per minute at 25 °C.

### 4.12. Evaluation of Apoptotic and Survival-Related Markers

Apoptosis- and survival-related proteins, including Bax, Bcl-2, caspase-3, cytochrome c, p53, and phosphorylated AKT (p-AKT), were quantified in colon tissue using ELISA kits from MyBioSource. Catalog numbers were MBS3807583 (Bax), MBS2515143 (Bcl-2), MBS261814 (caspase-3), MBS165286 (cytochrome c), MBS453009 (p53), and MBS1600201 (p-AKT). Procedures were performed according to the manufacturers’ protocols, ensuring uniform assay conditions. Absorbance was measured at 450 nm, and the resulting values were used to quantify the targeted proteins involved in apoptosis and in regulating cell survival.

### 4.13. Quantification of HMG-CoA Reductase

HMG-CoA reductase (HMGCR) levels in colon tissue were measured using a rat ELISA kit (MyBioSource, San Diego, CA, USA; Cat. No. MBS761708). The assay uses a capture antibody pre-coated on 96-well plates and a biotinylated detection antibody with streptavidin-HRP. Standards and samples were added and processed according to the manufacturer’s instructions. Color development with the TMB substrate was stopped with an acidic solution, and absorbance was measured at 450 nm. HMGCR concentrations were calculated from the standard curve and expressed as ng/mg protein.

### 4.14. Assessment of PCNA and Ki-67 Levels in Colonic Tissue

Colonic PCNA and Ki-67 were determined using rat sandwich ELISA kits (MyBioSource^®^, PCNA: Cat. No. MBS2515480; Ki-67: Cat. No. MBS705024). Samples and standards were applied to pre-coated plates, followed by detection antibodies and HRP-conjugated avidin. After incubation, washing, and substrate addition, the reaction was stopped, and absorbance was read at 450 ± 2 nm. Concentrations were calculated from standard curves and expressed as ng/mg protein.

### 4.15. RNA Isolation, cDNA Synthesis, and RT-qPCR Analysis

**Figure 16:**
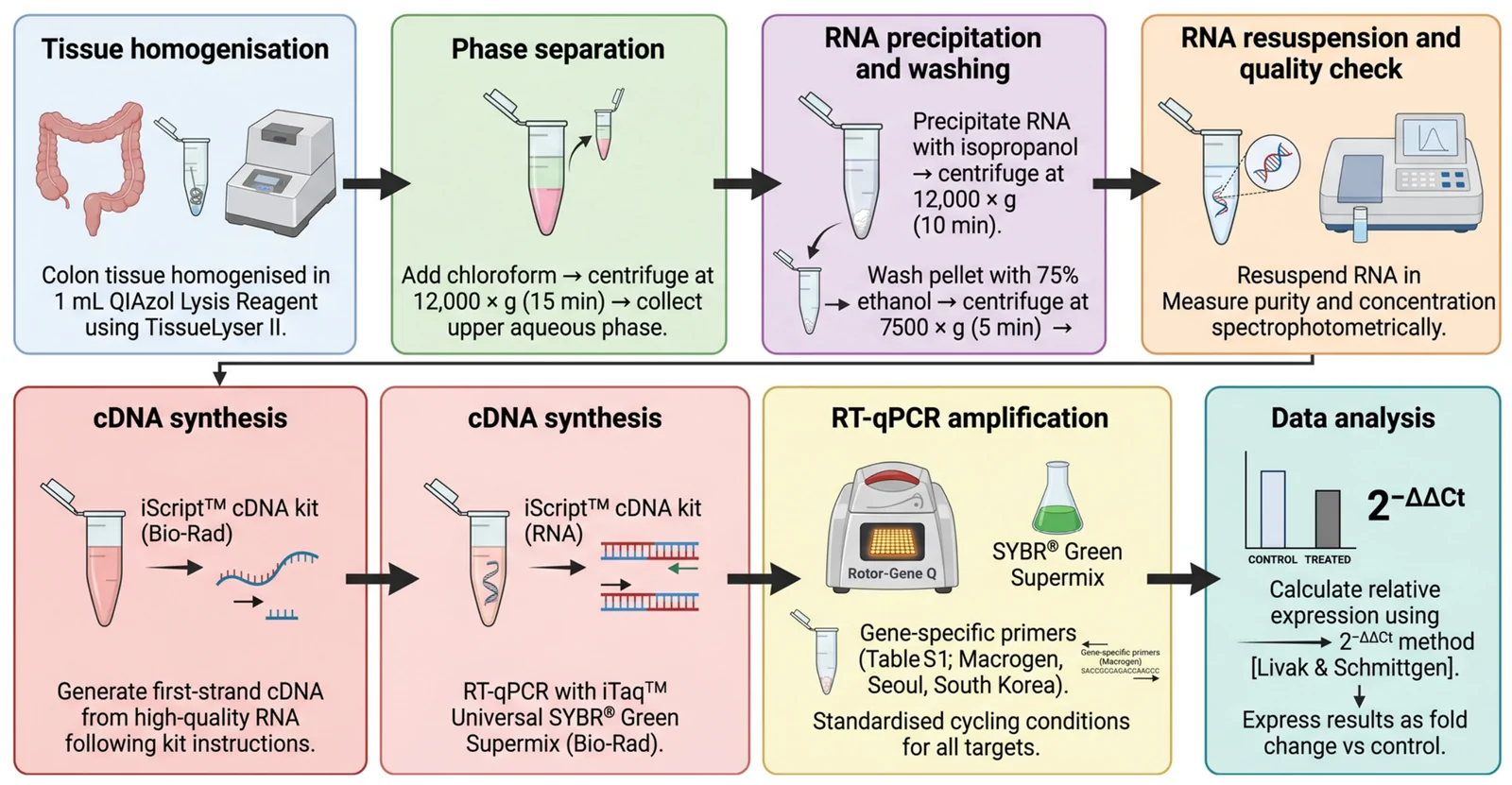
Workflow for RNA isolation, cDNA synthesis, and RT-qPCR analysis in colonic tissue. Colon samples are processed through QIAzol-based extraction, cDNA synthesis, and SYBR Green RT-qPCR using gene-specific primers, and relative expression is derived by the 2^−ΔΔCt^ method. It was generated with the assistance of “GAAbstract” (https://gaabstract.com/), accessed on 29 June 2026).

Total RNA was extracted from colonic tissue using a QIAzol Lysis Reagent (Qiagen GmbH, Hilden, Germany; Cat. No. 79306) according to the manufacturer’s instructions. Complementary DNA (cDNA) was synthesized using the iScript™ cDNA Synthesis Kit (Bio-Rad Laboratories, Hercules, CA, USA; Cat. No. 1708891). Quantitative real-time PCR was performed using iTaq™ Universal SYBR^®^ Green Supermix (Bio-Rad Laboratories, Hercules, CA, USA; Cat. No. 172-5121) on a Rotor-Gene Q real-time PCR system (Qiagen GmbH, Hilden, Germany) as outlined in the workflow diagram (Figure 16). Gene-specific primers are listed in Table S1, and relative expression was calculated [69]. All quality control measures were run according to MIQE 2 guidelines [70].

### 4.16. Histological Examination

Immediately after excision, colon tissues were fixed in 10% neutral buffered formalin at a 20:1 (fixative: tissue) ratio and kept at room temperature for 72 h. Fixed samples were dehydrated through a graded ethanol series (70–100%), cleared in xylene, and embedded in paraffin wax at 60 °C. Paraffin blocks were sectioned at 4–5 µm thickness using a rotary microtome, and sections were mounted on glass slides. Deparaffinization was carried out in xylene, followed by rehydration through descending ethanol grades (100–70%) and rinsing in distilled water. Sections were stained with hematoxylin and eosin, then dehydrated, cleared in xylene, and mounted. Histological features were examined under a light microscope.

### 4.17. Immunohistochemical Assay of NRF2 and NF-κB

Paraffin-embedded colon sections were deparaffinized in xylene and rehydrated through graded ethanol to distilled water. Antigen retrieval was achieved by heating in 0.01 M citrate buffer (pH 6.0) for 10 min in a microwave oven. Endogenous peroxidase activity was blocked with 3% hydrogen peroxide in methanol for 10 min, and non-specific binding was reduced by incubation with 5% bovine serum albumin (BSA) for 30 min at room temperature.

Sections were incubated overnight at 4 °C with primary antibodies against NRF2 (rabbit polyclonal, 1:200; Abcam, Cambridge, UK; ab62352) and NF-κB p65 (rabbit monoclonal, 1:200; Abcam, ab32536). After washing with PBS, sections were incubated with biotinylated goat anti-rabbit secondary antibody (1:500; Abcam) for 30 min, followed by streptavidin–HRP for 20 min. Immunoreactivity was visualized with 3,3′-diaminobenzidine (DAB), producing a brown precipitate. Sections were counterstained with Mayer’s hematoxylin, dehydrated, cleared in xylene, and mounted with DPX. Slides were examined at ×400 magnification, and five randomly selected fields per section were captured using a digital imaging system. Immunopositive areas were quantified using ImageJ version 1.54 (National Institutes of Health (NIH), open source, Bethesda, MD, USA).

For histopathological/immunohistochemical assessments, slides were coded before evaluation, and the pathologist performing qualitative scoring and quantitative image analysis was not informed of the group allocation at the time of assessment. Thus, histology and immunohistochemistry analyses were conducted in a blinded manner with respect to treatment groups.

### 4.18. Statistical Analysis

Raw data were collated and checked for entry errors in Microsoft Excel prior to analysis. The distribution of each variable was examined with the Shapiro–Wilk test, and equality of variances across groups was assessed using Levene’s test. Group comparisons were then performed using one-way analysis of variance (ANOVA) with PROC ANOVA in SAS/STAT software, version 9.4 (SAS Institute Inc., Cary, NC, USA). When the overall ANOVA indicated significant differences among groups, Tukey’s honestly significant difference (HSD) test was used to identify specific pairwise comparisons. Results are reported as mean ± standard error of the mean (SEM), and a two-sided *p*-value below 0.05 was interpreted as statistically significant. Graphical representations of the data were produced with GraphPad Prism (version 9.0, GraphPad Software, Boston, MA, USA).

## 5. Conclusions

In summary, DMH exposure in rats was associated with marked metabolic, oxidative, inflammatory, proliferative, apoptotic, autophagy-related, and histopathological alterations in colonic tissue. Free MOR attenuated several of these DMH-associated changes, whereas MOR-Lips were generally associated with more pronounced improvement in the measured biochemical, molecular, and histopathological profiles. These findings indicate that liposomal delivery may enhance morin’s biological activity in this experimental setting. However, because quantitative preneoplastic and neoplastic endpoints, including aberrant crypt foci, tumor incidence, tumor multiplicity, and tumor burden, were not assessed, the present results do not establish inhibition of colorectal carcinogenesis or chemopreventive efficacy. Further studies should include these disease-relevant endpoints, together with pharmacokinetic and biodistribution analyses, repeated-dose safety evaluation, validation in complementary CRC models, and mechanistic interventions to clarify causal pathway involvement and translational relevance.

## Figures and Tables

**Table 1:** Changes in particle size, polydispersity index, zeta potential, and morin retention of MOR-Lips during storage at 4 °C for 3 months.

Storage Time	Particle Size (nm)	PDI	Zeta Potential (mV)	Morin Retention (%)
Month 0	65.1 ± 1.8	0.182 ± 0.012	−47.1 ± 1.5	100.0 ± 0.0
Month 1	66.0 ± 1.9	0.187 ± 0.011	−46.8 ± 1.5	98.9 ± 1.1
Month 2	67.1 ± 2.1	0.193 ± 0.014	−46.2 ± 1.7	97.5 ± 1.3
Month 3	68.3 ± 2.2	0.201 ± 0.015	−45.4 ± 1.8	96.2 ± 1.4

PDI: polydispersity index.

## Data Availability

The original contributions presented in this study are included in the article/Supplementary Material. Further inquiries can be directed to the corresponding authors.

## Supplementary Materials

The following supporting information can be downloaded at: https://www.mdpi.com/article/10.3390/ph19091400/s1, Table S1: Primers for qRT-PCR analysis of gene expression in rat colonic tissues.

## Abbreviations

The following abbreviations are used in this manuscript:

AKT	Protein kinase B
Bax	Bcl-2–associated X protein
BCL-2	B-cell lymphoma 2
CA19-9	Carbohydrate antigen 19-9
CA125	Carbohydrate antigen 125
CAT	Catalase
CEA	Carcinoembryonic antigen
COX-2	Cyclooxygenase-2
CRC	Colorectal cancer
Cyt-c	Cytochrome c
DAB	3,3′-Diaminobenzidine
DMH	1,2-Dimethylhydrazine
DL	Drug loading
DLS	Dynamic light scattering
DTNB	5,5′-Dithiobis(2-nitrobenzoic acid)
EE	Encapsulation efficiency
ELISA	Enzyme-linked immunosorbent assay
FTIR	Fourier transform infrared spectroscopy
GSH	Reduced glutathione
HMG-CoA	3-Hydroxy-3-methylglutaryl coenzyme A
HMGCR	HMG-CoA reductase
HO-1	Heme oxygenase-1
HR-TEM	High-resolution transmission electron microscopy
IHC	Immunohistochemistry/immunohistochemical
IL-1β	Interleukin-1 beta
IL-6	Interleukin-6
LC3	Microtubule-associated protein 1 light chain 3
LC3-II	Lipidated form of LC3 (LC3-phosphatidylethanolamine conjugate)
MDA	Malondialdehyde
MOR	Morin
MOR-Lips	Morin-loaded liposomes
MPO	Myeloperoxidase
NF-κB	Nuclear factor kappa B
NO	Nitric oxide
NRF2	Nuclear factor erythroid 2–related factor 2
PBS	Phosphate-buffered saline
PCNA	Proliferating cell nuclear antigen
PDI	Polydispersity index
PGE2	Prostaglandin E2
p-AKT	Phosphorylated AKT
p62	Sequestosome 1 (SQSTM1)
qPCR/RT-qPCR	Quantitative real-time polymerase chain reaction
ROS	Reactive oxygen species
RNS	Reactive nitrogen species
SEM	Standard error of the mean
SOD	Superoxide dismutase
TBARS	Thiobarbituric acid reactive substances
TEM	Transmission electron microscopy
TLR4	Toll-like receptor 4
TNF-α	Tumor necrosis factor-α
p53	Tumor protein p53
8-OHdG	8-Hydroxy-2′-deoxyguanosine
