# Supplementary material for: Liposomal Morin Attenuates DMH-Associated Colonic Oxidative Stress, Inflammation, and Apoptosis/Autophagy-Related Dysregulation in Rats
Source: Pharmaceuticals (Basel). 2026 Sep 4;19(9):1400. doi: 10.3390/ph19091400 (PMC13610203; doi:10.3390/ph19091400)
Supplement: Supplementary file 1 [file pharmaceuticals-19-01400-s001.zip › pharmaceuticals-4480161-supplementary.pdf]

Liposomal Morin Attenuates DMH-Associated Colonic Oxidative Stress, Inflammation, and Apoptosis/Autophagy-Related Dysregulation in Rats

Table S1. Primers for qRT-PCR analysis of gene expression in rat colonic tissues.

Gene	Sequences (5′-3′)	Accession no	Length (bp)
Nrf2	F: TTTGTAGATGACCATGAGTC<br>R: TCCTGCCAAACTTGCTCCAT	NM_031789.2	161
TLR4	F: ATCATCCAGGAAGGCTTCCA<br>R: GCTGCCTCAGCAAGGACTTC	NM_019178.2	120
NF-κB	F: AGTCCCGCCCCTTCTAAAAC<br>R: CAATGGCCTCTGTGTAGCCC	NM_001276711.1	106
P62	F: CTAGGCATCGAGGTTGACATT<br>R: CTTGGCTGAGTACCACTCTTA	NM_017134.2	150
LC3	F: GGTCCAGTTGTGCCTTTATTGA<br>R: GTGTGTGGGTTGTGTACGTCG	NM_022867	152
Beclin-1	F: CAACCCCATGCTGTCCTTTC<br>R: CTCTTGTCCCTTCCCCACAT	NM_053739	131
β-Actin	F: CAGCCTTCCTTCTTGGGTATG<br>R: AGCTCAGTAACAGTCCGCCT	NM_031144.3	360

Nrf2: nuclear factor erythroid 2–related factor 2; TLR4: Toll-like receptor 4; NF-κB: nuclear factor kappa B; p62 (SQSTM1): sequestosome 1, a selective autophagy adaptor protein involved in cargo recognition and degradation. LC3: microtubule-associated protein 1 light chain 3, a key marker of autophagosome formation. Beclin-1: autophagy-related protein essential for autophagosome initiation. β-Actin: housekeeping gene used as an internal control for data normalization.
